# Discovery of Small Molecules that Inhibit the Disordered Protein, p27^**Kip1**^

**DOI:** 10.1038/srep15686

**Published:** 2015-10-28

**Authors:** Luigi I. Iconaru, David Ban, Kavitha Bharatham, Arvind Ramanathan, Weixing Zhang, Anang A. Shelat, Jian Zuo, Richard W. Kriwacki

**Affiliations:** 1 Department of Structural Biology, Memphis, TN 38105; 2 Department of Developmental Neurobiology, Memphis, TN 38105; 3 Department of Chemical Biology and Therapeutics, St. Jude Children’s Research Hospital, Memphis, TN 38105; 4 Computational Science and Engineering Division, Health Data Sciences Institute, Oak Ridge National Laboratory, Oak Ridge, TN 37830; 5 Department of Microbiology, Immunology and Biochemistry, The University of Tennessee Health Science Center, Memphis, TN 38163.

## Abstract

Disordered proteins are highly prevalent in biological systems, they control myriad signaling and regulatory processes, and their levels and/or cellular localization are often altered in human disease. In contrast to folded proteins, disordered proteins, due to conformational heterogeneity and dynamics, are not considered viable drug targets. We challenged this paradigm by identifying through NMR-based screening small molecules that bound specifically, albeit weakly, to the disordered cell cycle regulator, p27^Kip1^ (p27). Two groups of molecules bound to sites created by transient clusters of aromatic residues within p27. Conserved chemical features within these two groups of small molecules exhibited complementarity to their binding sites within p27, establishing structure-activity relationships for small molecule:disordered protein interactions. Finally, one compound counteracted the Cdk2/cyclin A inhibitory function of p27 *in vitro*, providing proof-of-principle that small molecules can inhibit the function of a disordered protein (p27) through sequestration in a conformation incapable of folding and binding to a natural regulatory target (Cdk2/cyclin A).

Intrinsically disordered proteins (IDPs) are highly prevalent in eukaryotic cells and often perform regulatory and signaling functions^1^. As a structural class, IDPs are more tightly regulated than folded proteins from yeast to humans^2^ and, upon overexpression, are more prone to alter cell phenotype than their folded counterparts^3^. Thus, maintaining normal disordered protein homeostasis is critical for cellular behavior. The phenotypes associated with overexpression or hyperactivity of proteins with folded domains can be counteracted by small molecule inhibitors of, for example, enzyme function (*e.g.,* Gleevec which inhibits BCR-Abl in chronic myelogenous leukemia^4^) or protein-protein interactions (*e.g.,* ABT-263 and ABT-199 which inhibit BCL-2 and BCL-xL in hematological and lymphoid malignancies^5^,^6^). In contrast, disordered proteins represent challenging targets for inhibition by small molecules due to their dynamic and heterogeneous conformations. Nonetheless, progress has been made. For example, an inhibitor of the phosphatase PTP1B (MSI-1436), with known roles in diabetes, obesity, and breast cancer, was recently shown through structural, biochemical and functional assays to act through an allosteric mechanism by binding to a disordered regulatory region of the enzyme^7^. Also, a small molecule (YK-4-279) that binds to the disordered EWS-FLI1 fusion oncoprotein associated with Ewing’s sarcoma family tumors inhibited direct binding to RNA helicase A (RHA)^8^, a functional partner of EWS-FLI1 in tumor cells^8^, and altered RHA-dependent protein interactions and RNA splicing^9^. Both of these compounds (MSI-1436 and YK-4-279) exhibited on-target effects in cellular assays^8^,^9^. Other studies have identified small molecules that target disordered cMyc^10^,^11^,^12^, α-synuclein^13^, and Alzheimer β-amyloid peptide^14^. A recent computational study^15^ showed that a small molecule (10074-A4) previously reported to modulate cMyc function^10^ bound in different ways to different cMyc molecules within an ensemble of many disordered conformations, leading the authors to suggest the concept of “ligand clouds binding to protein clouds”. In the studies discussed above, small molecules that target disordered proteins were discovered using a variety of approaches, including functional screens, *in vitro* binding screens and/or computational screens. Nuclear magnetic resonance (NMR)-based screening of low molecular weight small molecules termed fragments (reviewed in^16^) binding to folded protein targets is a well-established method for identifying initial “hits” in the process of drug discovery^17^,^18^. However, NMR-based fragment screening has not, to our knowledge, been applied to identify small molecules that bind to a disordered protein target. Here, we utilized NMR-based screening to identify fragment molecules that bind to and modulate the function of the prototypical disordered protein, p27^Kip1^ (p27; also known as CDKN1B), a regulator of the cyclin-dependent kinases that control eukaryotic cell division^19^.

The motivation for targeting p27 was two-fold. First, the structural and functional features of p27 are well understood^20^,^21^,^22^,^23^, providing an ideal model system for studying small molecule:disordered protein interactions. Second, the ability to chemically modulate p27 function would be beneficial in several biological settings. For example, p27 is inappropriately phosphorylated in breast cancer on threonine 157, which is associated with abnormal cytoplasmic localization and up-regulation of cell migration^24^,^25^,^26^,^27^. The availability of a small molecule inhibitor of p27 would be beneficial to prevent abnormal migration of breast cancer cells. Alternatively, in both sensory and non-sensory epithelial cells of the inner ear, p27 maintains cell cycle exit and terminal differentiation^28^ and its inhibition resulted in their cell cycle reentry and regeneration for hearing restoration^29^,^30^. While small molecules that inhibit the transcription of p27 have been reported^31^, here we developed approaches to identify small molecules that bind directly to p27 and have potential to alter its function in the two cellular settings discussed above.

The target of our studies was the N-terminal, kinase inhibitory domain of p27 (p27-KID), which binds to and regulates the catalytic activity of nuclear cyclin-dependent kinase (Cdk)/cyclin complexes that control eukaryotic cell division^32^. p27-KID, which is highly disordered in isolation^20^,^21^, adopts an extended conformation upon binding to Cdk2/cyclin A (Fig. 1a) that can be subdivided into three, functionally distinct sub-domains. Sub-domain D1 binds to a conserved pocket on cyclin A and blocks substrate recruitment^33^; sub-domain D2 forms intra- and inter-molecular (between p27 and Cdk2) β-strands upon binding to Cdk2 and also inserts a turn of helix into its ATP binding pocket, inhibiting kinase activity^34^; and sub-domain LH forms an α-helix that connects sub-domains D1 and D2. We hypothesized that, if small molecules that bind to p27-KID could be identified, they may induce the disordered polypeptide to adopt conformations that are incompetent for binding to Cdk/cyclin complexes. We tested this hypothesis by screening a library of fragment molecules for binding to p27-KID using NMR spectroscopy. We identified two subsets of fragment molecules (36 in total) that differentially bound weakly but specifically to two partially overlapped regions of p27-KID. From these subsets, we then generated pharmacophore models that allowed identification of additional small molecules that bound to p27-KID and further clarification of structure-activity relationships. A variety of assays, including fluorescence anisotropy, NMR spectroscopy and a Cdk2 kinase activity assay, were used to demonstrate that one of the identified small molecules displaced the kinase binding region of p27 from Cdk2 and partially restored catalytic activity of Cdk2. In addition, molecular dynamics computations provided insights into the dynamic “structure” of the region of p27 targeted by small molecules. Our results provide insights into the nature of interactions between small molecules and a disordered protein and demonstrate that such interactions can alter disordered protein regulatory function.

## Results

### Discovery of small molecules that bind specifically to p27-KID

**Figure 1:**
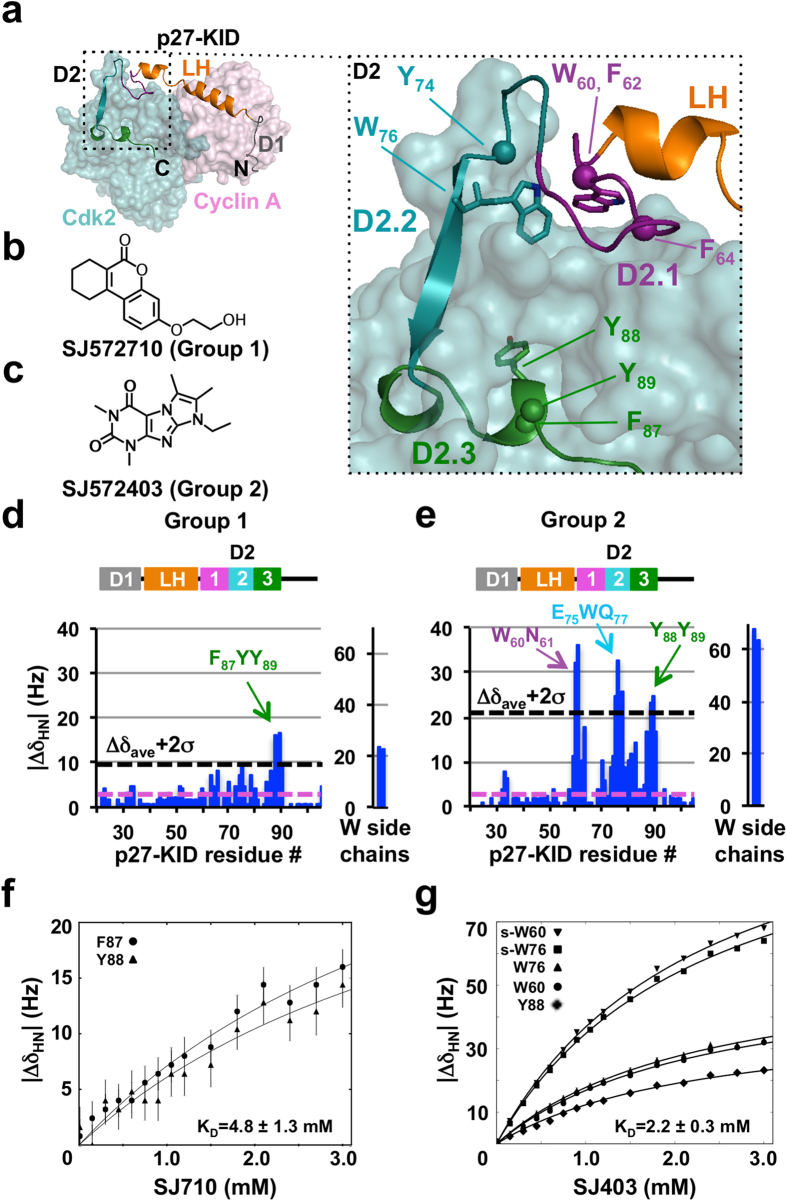
Identification of two groups of small molecules that bind specifically to distinct but overlapping regions of disordered p27-KID. (**a**) Structure of p27-KID bound to Cdk2/Cyclin A (PDB ID 1JSU); the sub-domains of p27-KID, including D1, LH, D2.1, D2.2, and D2.3, are indicated. (**b**,**c**) Chemical structures of two small molecules, SJ572710 (SJ710) (**b**) and SJ572403 (SJ403) (**c**), that bound to p27-KID and are members of Group 1 and Group 2, respectively (see text for Group definitions). (**d**,**e**) Histograms showing individual ^1^H chemical shift perturbation values (through analysis of 2D ^1^H-^15^N “in-phase” HSQC spectra) from titrations of the compounds in (b) and (c), respectively, into ^15^N-p27-KID. The threshold for identifying specific interactions with p27-KID residues was defined as two standard deviations above the average of the perturbation values (represented by a dotted black line in the graph). The experimental spectral resolution in the ^1^H dimension (2.4 Hz) is represented by the dotted magenta line. Chemical shift perturbations for molar ratios of ^15^N-p27-KID (100 μM) to inhibitors of 1:30 are shown. (**f**,**g**) Amide proton chemical shift perturbations plotted versus the concentration of SJ710 (**f**) and SJ403 (**g**), respectively. Binding isotherms of select residues show specific binding between p27-KID and fragment hits. The trajectories of the chemical shifts (solid black lines) report global dissociation constants of 4.8 ± 1.3 and 2.2 ± 0.3 mM for the interactions of p27-KID with SJ710 (**f**) and SJ403 (**g**), respectively.

We used one-dimensional (1D) ^1^H WaterLOGSY^35^ and STD^36^ NMR methods to identify “fragment-like” small molecules^37^,^38^,^39^ that bound to p27-KID. Fragment molecules were selected from either a commercial library (1,100 compounds from the Ro3 collection, Maybridge/Thermo Fisher Scientific) or an in-house library of 1,222 compounds based on the “Rule of Three”^40^ and other criteria (see Methods). Two and seven molecules each were identified from the respective libraries to bind p27-KID (termed “hits”; representative 1D ^1^H NMR data is shown in Suppl. Fig. 1a,b and all preliminary hits are presented in Suppl. Table 1a). Binding sites for these compounds were identified by titration into ^15^N-p27-KID and analysis of two-dimensional (2D) ^1^H-^15^N HSQC spectra. Significant chemical shift perturbations (CSPs), which were largest for amide ^1^H resonances (see Methods), were only observed for amide groups of residues within the D2 sub-domain of p27-KID (p27-D2); eight hits caused CSPs within a short region with the sequence F_87_YY_89_ [(F, phenylalanine; and Y, tyrosine), termed sub-region D2.3; Fig. 1a,d] and one hit caused CSPs within the same region as well as within two other regions near residues W_60_N_61_ and E_75_WQ_77_ [(W, tryptophan; N, asparagine; E, glutamic acid; and Q, glutamine), termed sub-domains D2.1 and D2.2, respectively; Fig. 1a,e]. We termed these molecules Groups 1 and 2, respectively (Fig. 1b,c); representative ^1^H CSP histograms are shown in Fig. 1d,e, and ^15^N CSPs are presented in Suppl. Fig. 2. Data for all other small molecules are shown in Suppl. Fig. 3.

**Figure 2:**
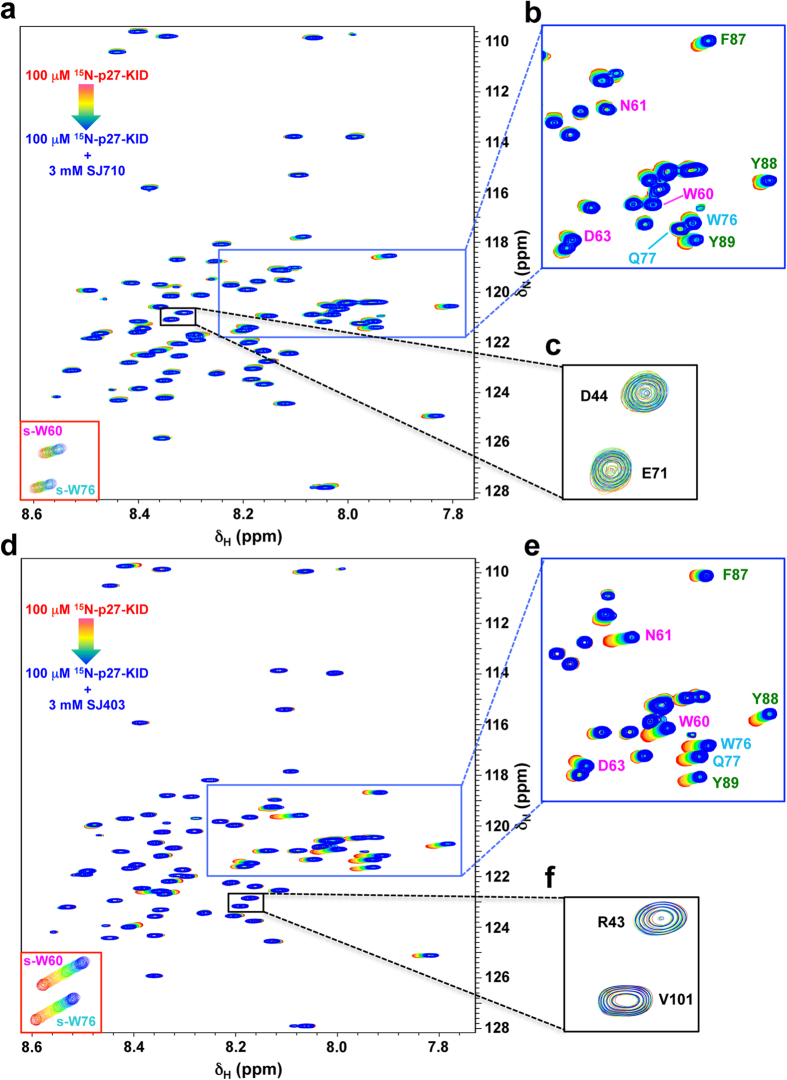
Fragment hits show specific interactions with specific regions within the D2 subdomain of p27-KID. Overlaid 2D ^1^H-^15^N “in-phase” HSQC NMR spectra showing chemical shift perturbations of specific residues within the D2 subdomain of p27-KID after titration of fragment hits SJ710 (**a**, Group 1) and SJ403 (**d**, Group 2). For each hit, a total of sixteen spectra were recorded. The concentration of ^15^N-p27-KID (100 μM) was kept constant throughout the titration and the concentration of the inhibitor was varied from 0 (red) to 3 mM (blue). All experiments were performed on a 800 MHz spectrometer and utilized acquisition parameters that provided a spectral resolution of 2.4 Hz and 1.7 Hz in the ^1^H and ^15^N dimensions, respectively. The insets in the lower left show resonances for the tryptophan indole NH moieties (s-W60, s-W76). (**b**,**e**) Expanded regions (blue box) showing a subset of the p27-KID residues involved in interaction with SJ710 (**b**) and SJ403 (**e**), respectively. (**c**,**f**) Expanded regions (black box) showing two of the p27-KID residues that do not show any perturbations upon interaction with SJ710 (**c**) and SJ403 (**f**), respectively.

We analyzed the Group 1 and 2 molecules using computational modeling and identified additional candidate p27-KID binding molecules in the two fragment libraries that were not detected by the original 1D NMR screens. 1D and 2D NMR analysis of these molecules led to the identification of 15 additional p27-D2-binding compounds (six with Group 1-like binding features and nine with Group 2-like features; Suppl. Fig. 3b and Suppl. Table 1b). CSPs were observed at high compound concentrations, consistent with relatively weak binding to p27-D2, but were specific to the noted regions within p27-D2 and rigorously reproducible. Sixteen-point 2D ^1^H-^15^N “in phase” HSQC NMR titrations of a Group 1 (SJ572710, hereafter termed SJ710) and 2 (SJ572403, hereafter termed SJ403) hits, respectively, into a constant concentration of ^15^N-p27-KID (100 μM), provided interpretable binding isotherms for specific resonances of p27-KID; the determined dissociation constant (K_d_) values were 4.8 ± 1.3 and 2.2 ± 0.3 mM, respectively (Fig. 1f,g). The overlaid 2D ^1^H-^15^N HSQC NMR spectra are displayed in Fig. 2.

### Chemical features of p27-D2-binding molecules

**Figure 3:**
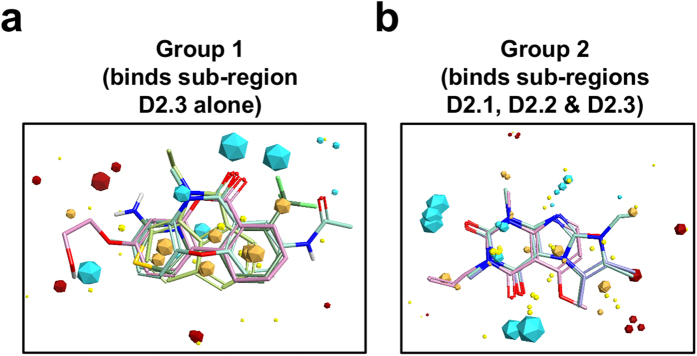
Consensus field maps for the two groups of small molecules that bound to p27-KID. Blue and red field points indicate where electropositive and electronegative groups are favored, respectively, in the protein binding partner; gold field points indicate regions where hydrophobic interactions are favored; and yellow field points indicate regions where favorable van der Waals contacts are possible. The size of the field point increases with the magnitude of the favorable interaction energy. Two representative molecules from Group 1 (**a**) and Group 2 (**b**) are shown aligned to their respective consensus field maps.

Three-dimensional (3D) molecular interaction field analysis^41^ (see Methods section) identified common chemical features of the two groups of p27-D2 binding molecules (Fig. 3a,b). This analysis revealed interaction “field points” around the small molecules that can favorably participate in electrostatic, van der Waals, and hydrophobic interactions. These field points are used to calculate molecular similarity and to align molecules, even those with different two-dimensional topologies, to define a common pharmacophore.

The molecules in Groups 1 and 2 have two or three heterocyclic aromatic rings but significantly differ in the distribution of favorable field points. Group 1 is mainly defined by a large hydrophobic core (multiple gold polygons in close proximity; Fig. 3a), one large electropositive interaction region (two cyan polygons in close proximity), and two smaller electropositive (cyan) and electronegative (red) field points at the opposite end of the hydrophobic core. In contrast, the field map for Group 2 (Fig. 3b) exhibited a smaller hydrophobic core relative to Group 1 and two equally-sized regions of favorable electropositive interaction. To expand the diversity of our chemical screen, we used the consensus field maps for Group 1 and 2 molecules to identify 184 additional possible p27-D2-binding molecules within a library of 10,455 commercially-available fragment-like molecules. 1D and 2D NMR analysis of these identified 12 additional p27-D2-binding compounds (8 with Group 1-like binding features and 4 with Group 2-like features; Suppl. Fig. 3c and Suppl. Table 1c). These additional molecules had CSP profiles comparable to the previously identified hits. To further test the specificity of small molecule:disordered protein interactions, we determined whether the simple amino acids tryptophan and tyrosine bound to p27-KID. However, even when titrated to a 30-fold molar excess, neither aromatic amino acid cause chemical shift perturbations in 2D HSQC spectra upon titration into p27-KID (Suppl. Fig. 4). This is most likely because they lack the specific chemical features embodied in the pharmacophore models for the two groups of fragment hits.

### A Group 2 molecule modulates p27’s Cdk regulatory function

**Figure 4:**
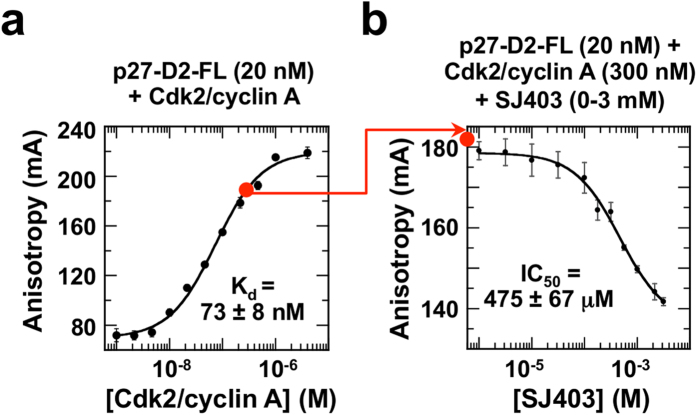
Fluorescence anisotropy analysis of displacement of p27-D2-FL from Cdk2/cyclin A by SJ403. (**a**) Fluorescence anisotropy analysis of Cdk2/cyclin A binding to p27-D2-FL. The starting condition (20 nM p27-D2-FL, 300 nM Cdk2/cyclin A) for assessing the effect of fragment hit SJ403 on p27 function is represented as the red circle on the binding isotherm. (**b**) Titration of SJ403 (0–3 mM) caused the displacement of p27-D2 from Cdk2/cyclin A, resulting in a decrease in fluorescence anisotropy values. Experiments were performed in triplicate and average values and the standard deviations of the means are shown.

We used the Group 2 molecule, SJ403 (Suppl. Table 1b), to test our hypothesis that molecules that bind to p27 can alter its Cdk regulatory function. For these experiments, because SJ403 binds weakly to p27-KID, we studied its ability to modulate the binding of p27-D2 to Cdk2/cyclin A (K_d_ value binding to Cdk2/cyclin A, 73 ± 8 nM; Fig. 4a *versus* 5 nM for p27-KID^20^). We first used fluorescence anisotropy (FA) to monitor displacement of a single-cysteine (Cys) mutant of p27-D2, with arginine 93 mutated to Cys (R_93_C) and labeled with Alexa Fluor 488 (p27-D2-FL), from Cdk2/cyclin A by SJ403. Titration of SJ403 caused concentration-dependent reduction of FA of p27-D2-FL, with an IC_50_ value of 475 ± 67 μM (Fig. 4b), suggesting that SJ403 displaced p27-D2 from Cdk2/cyclin A.

**Figure 5:**
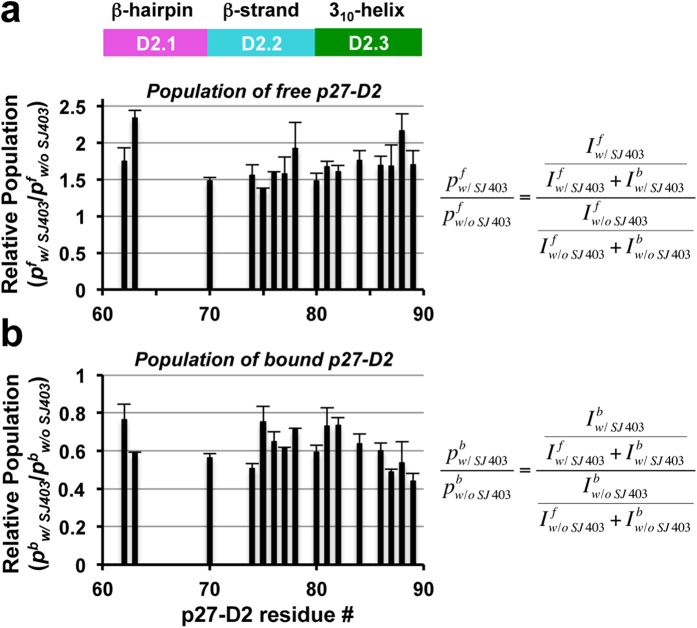
2D ^1^H-^15^N TROSY-HSQC NMR analysis of displacement of ^15^N-p27-D2 from Cdk2/cyclin A by SJ403. 2D ^1^H-^15^N TROSY-HSQC NMR spectra of p27-D2/Cdk2/cyclin A in the absence (control) and presence of SJ403 (3 mM), respectively, were recorded. For the residues reported, both free and bound p27-D2 resonances were observed in the TROSY-HSQC spectra. An increase in the relative intensity of free p27-D2 resonances was observed in the TROSY-HSQC spectrum of p27-D2/Cdk2/cyclin A in the presence of SJ403, with respect to the control spectrum (**a**), while the resonances of p27-D2 bound to Cdk2/cyclin A showed a decrease in relative intensity (**b**). This permits the calculation of populations of free (*f*) and bound (*b*) p27-D2, in the absence (*w/o SJ403*) and presence of SJ403 (w/*SJ403*)). The p^f^_w/o SJ403_ values represent the relative populations of resonances for free p27-D2 before addition of SJ403 and the p^f^_w/SJ403_ values are the relative populations of free state p27-D2 resonances after SJ403 addition. Correspondingly, the p^b^_w/o SJ403_ values represent the relative populations of resonances for p27-D2 bound to Cdk2/cyclin A before addition of SJ403 and the p^b^_w/SJ403_ values are the relative populations of bound state p27-D2 resonances after SJ403 addition. The mean values and standard deviations of the means for triplicate measurements are shown; one set of triplicate 2D ^1^H-^15^N TROSY-HSQC spectra are illustrated in Suppl. Fig. 7.

We next used NMR spectroscopy to monitor the displacement of ^2^H/^13^C/^15^N-labeled p27-D2 from Cdk2/cyclin A by SJ403; the complex of p27-D2 with Cdk2/cyclin A (100 μM) was prepared with a slight excess of ^2^H/^13^C/^15^N-p27-D2 (mole ratio, 1.1:1.0 p27-D2:Cdk2/cyclin A). Peaks for unbound p27-D2 dramatically increased in intensity in the presence of SJ403, while the resonances for p27-D2 bound to Cdk2/cyclin A were reduced in intensity (Fig. 5 and Suppl. Fig. 7a–c), consistent with partial displacement of p27-D2 from Cdk2/cyclin A. A ratiometric method was used to analyze the 2D TROSY-HSQC NMR spectra. In this method, the relative population of the free state resonances (p^f^; for the resonance of each p27-D2 residue) was determined as a fraction of the total intensity of both free and bound resonances for a given residue. These values were compared by forming the ratio of the two relative free state populations (p^f^_w/SJ403_/p^f^_w/o SJ403_); values greater than 1 indicated compound-dependent displacement. The relative populations for the bound state (p^b^) were also determined for samples without (p^b^_w/o SJ403_) and with SJ403 (p^b^_w/SJ403_). In this case, ratios less than 1 indicated displacement of p27-D2 from Cdk2/cyclin A. Furthermore, the chemical shift values of the free state resonances reflected binding of p27-D2 to SJ403 (Suppl. Fig. 7d,e), further supporting the conclusion that SJ403 shifts the binding equilibrium between p27-D2 and Cdk2/cyclin A to increase the population of unbound p27-D2 through protein:small molecule interactions.

**Figure 6:**
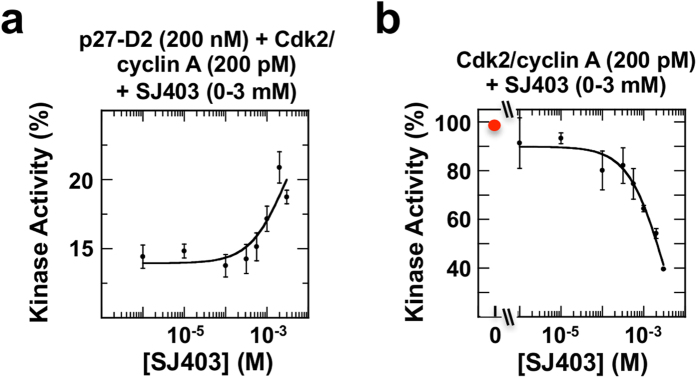
The small molecule SJ403 displaces p27-D2 from Cdk2/cyclin A and partially restores Cdk2 kinase activity. Analysis of Cdk2 kinase activity [within the Cdk2/cyclin A complex (200 pM) using Histone H1 as the substrate] in the presence of p27-D2 (200 nM) and varied concentrations of SJ403 (from 10 μM to 3 mM) (**a**) and varied concentrations of SJ403 alone (**b**). Cdk2 is partially active under the starting conditions in (a) and activity is enhanced through addition of SJ403. In the absence of p27-D2 (b), addition of SJ403 is associated with Cdk2 inhibition. All experiments were performed in triplicate and average values and the standard deviations of the means are shown.

The FA and NMR results indicated that SJ403 partially displaced p27-D2 from Cdk2/cyclin A; therefore, we next investigated whether displacement modulated the kinase activity of Cdk2. p27-D2, while lacking the D1 and LH sub-domains found in p27-KID, is still a moderately potent inhibitor of Cdk2/cyclin A (IC_50_ value, 152 ± 39 nM; Suppl. Fig. 9a). Under conditions wherein Cdk2/cyclin A was almost completely inhibited by p27-D2 (~13% of full kinase activity was maintained), titration of SJ403 over the concentration range shown in FA and NMR experiments to cause p27-D2 displacement led to increased kinase activity (from 13% to ~20%, a >50% increase; Fig. 6a and Suppl. Fig. 9b), consistent with partial displacement of p27-D2 from Cdk2/cyclin A by SJ403. Furthermore, in the absence of p27-D2, SJ403 substantially inhibited Cdk2/cyclin A activity at concentrations that, in the presence of p27-D2, were associated with p27-D2 displacement and increased kinase activity (Fig. 6b and Suppl. 9c). Thus, we conclude that the primary effect of SJ403 is displacement of p27-D2 from Cdk2/cyclin A (through the binding of SJ403 to p27-D2) and partial restoration of kinase activity, even as a secondary effect of SJ403 is to inhibit kinase activity (through the binding of SJ403 to Cdk2/cyclin A). These results provide proof-of-principle that a small molecule (SJ403) inhibits the function of a disordered protein (p27-D2) through sequestration in a conformation incapable of binding and inhibiting Cdk2/cyclin A.

### Molecular basis of recognition of p27 by small molecules

**Figure 7:**
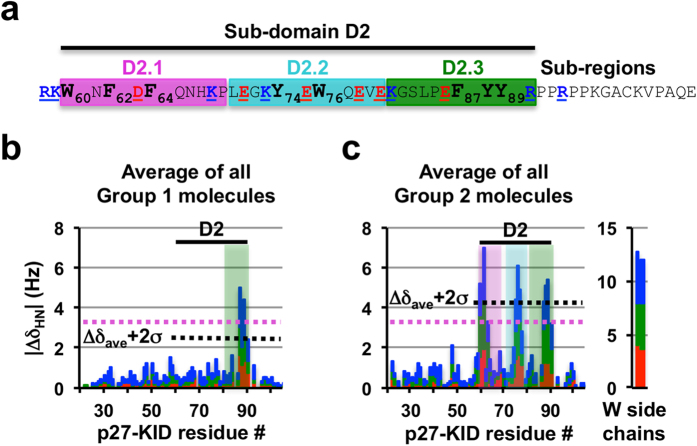
Small molecules bind to clusters of aromatic residues with the D2 sub-domain of p27-KID. (**a**) The amino acid sequence of p27-D2 showing sub-domain D2 and the individual small molecule binding regions (labeled D2.1, D2.2 and D2.3). The aromatic amino acids within these regions are illustrated in bold. These represent eight of the nine aromatic residues within p27-KID. (**b**,**c**) Average ^1^H chemical shift perturbation values for all Group 1 (**b**) and Group 2 (**c**) compounds illustrating preferences for interactions with Y (**b**) and W and Y (**c**) residues, respectively.

We previously showed based on NMR and molecular dynamics (MD) computational data that p27-KID exhibits different types of partially populated secondary structure in the free state, including helical structure within the LH sub-domain, a β-hairpin conformation within sub-region D2.1 and helical structure within sub-region D2.3 of sub-domain D2 (ref. 21 ; see Fig. 1a for sub-domain/sub-region nomenclature). Interestingly, the Group 1 and 2 molecules variably interacted with two of these partially structured regions of p27-KID (sub-regions D2.1 and D2.3), but also with sub-region D2.2, which is highly dynamic and does not exhibit persistent secondary structure^21^ (Fig. 7a–c); these interaction sites are all within the Cdk2-binding D2 sub-domain. Notably, the p27 binding molecules did not interact with sub-domains D1 or LH. Importantly, each of the regions within p27-KID that interacted with small molecules contained several aromatic amino acids (Fig. 7a). In fact, sub-domain D2 contains eight of the nine aromatic amino acids found within p27; a ninth, F_33_, is found within sub-domain D1 but did bind to small molecules. Group 1 molecules caused the largest CSPs for residues F_87_, Y_88_ and Y_89_ within sub-region D2.3 and Group 2 molecules perturbed resonances for these residues as well as for residues W_60_, N_61_ (sub-region D2.1) and E_75_, W_76_ and Q_77_ (sub-region D2.2). Phenylalanine (F) or tyrosine (Y) residues flank the tryptophan (W) residues, but exhibited smaller CSP values in the presence of Group 2 compounds than did the W residues, suggesting that the small molecules preferentially perturbed the electronic environment of the indole rings of these residues. We tested this hypothesis through mutagenesis of W_60_ or W_76_, or both, within p27-KID to either F or alanine (A) and used 2D NMR to map binding to SJ403. The singly mutant p27-KID variants exhibited patterns of CSPs similar to those observed for wild-type p27-KID except that perturbations near the mutated W residues were absent (Suppl. Fig. 5a,b,d,e). However, the two doubly mutated p27-KID variants exhibited only very weak CSPs within the F_87_YY_89_ aromatic cluster (Suppl. Fig. 5c,f). Together, these results indicated that each of the two W residues, W_60_ and W_76_, contributed substantially to binding to SJ403. Mutations of F_87_, Y_88_ or Y_89_ to A were associated with substantially reduced CSPs within the Group 1 compound binding region of p27-KID (Suppl. Fig. 5j–l), whereas mutation of R_90_ to A (Suppl. Fig. 5m) had little effect on the binding, implying that the clustering of the aromatic side chains is critical to the small molecule:protein interaction. With the Y_88_ to A p27-KID mutant, the Group 2 compound, SJ403, caused CSPs near the two W residues (W_60_ or W_76_) but not within the F_87_A_88_Y_89_ region of p27-KID (Suppl. Fig. 5h). However, with the F_87_ to A and Y_89_ to A mutants, SJ403 caused CSP patterns similar to that observed with wild-type p27-KID (Suppl. Fig. 5g,i), suggesting that Y_88_ contributes to a greater extent to interactions with SJ403 (and possibly other Group 2 compounds) than does Y_89_.

**Figure 8:**
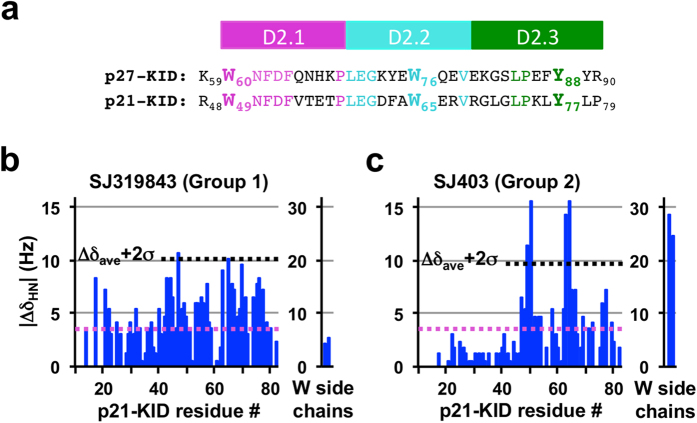
Interaction of p21-KID with representative p27 fragment hits. (**a**) Comparison of amino acid sequences of the D2 subdomains of p27-KID and p21-KID; (**b**,**c**) Individual ^1^H (Δδ_HN_) chemical shift perturbations (through analysis of 2D ^1^H-^15^N HSQC NMR spectra) caused by addition of SJ319843 (**b**, Group 1) and SJ403 (**c**, Group 2), respectively, to ^2^H/^15^N-p21-KID (20 μM). The threshold for identifying specific binding interactions with the p27-KID residues was defined as two standard deviations above the average of chemical shift perturbations (Δδ_ave_+2σ, represented as the dotted black line) and considered if perturbations were larger than the experimental ^1^H digital resolution (shown as dotted magenta line).

The two W residues and Y_88_ of p27 are conserved in the related disordered cell cycle regulatory protein, p21^Waf1/Cip1 ^42 (p21; Fig. 8a), allowing their dominance in interactions with Group 1 & 2 compounds to be further tested (Fig. 8b,c). The Group 2 compound, SJ403, interacted with residues near W_49_ and W_65_ of p21 (within the p21 kinase inhibitory domain, p21-KID) but not with Y_77_ (homologous to Y_88_ of p27; Fig. 8c), supporting the importance of W residues for interactions with this small molecule but also suggesting that clustering of at least two Y and F residues is required for additional interactions. The two leucine (L) residues flanking Y_77_ in p21 (L_76_ and L_78_) do not substitute for F_87_ and Y_89_ in p27. Similarly, the L_76_YL_78_ region of p21 does not support binding to a Group 1 compound (SJ319843; Fig. 8b). These findings with p21-KID further strengthen our explanation of the molecular basis for interactions of Group 1 and 2 compounds with p27.

**Figure 9:**
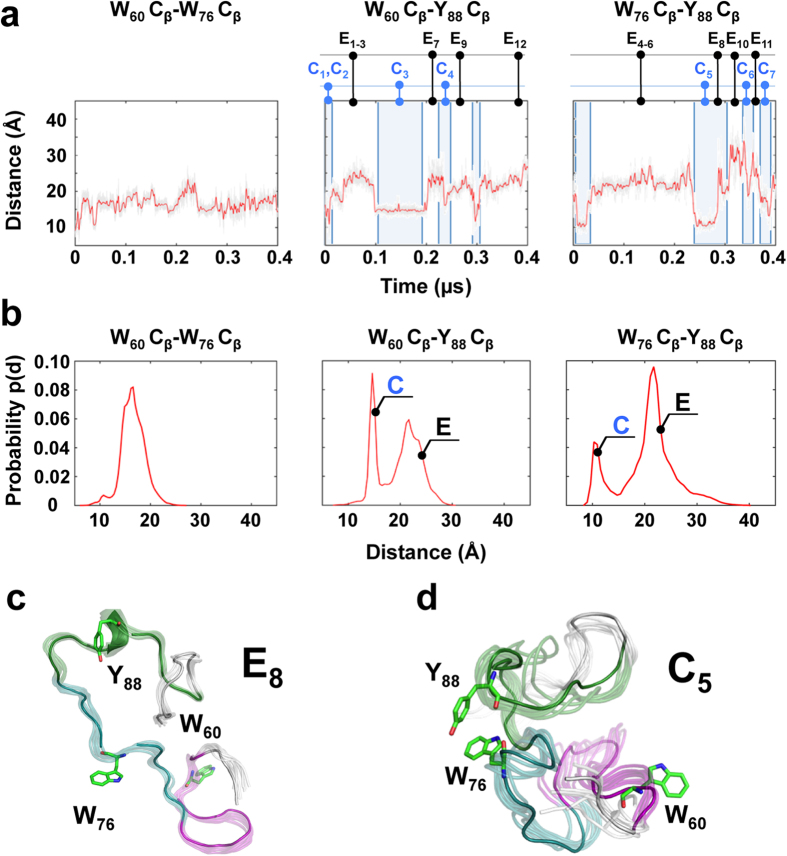
Distance profiles of W_60_, W_76_ and Y_88_ C^β^ atoms monitored as a function of time from MD simulations reveal extended and compact states in the p27-D2 conformational ensemble. (**a**) Distance between W_60_-W_76_ (left), W_60_-Y_88_ (center), and W_76_-Y_88_ (right) C^β^ atoms monitored over time from MD with gray lines representing the instantaneous distance (every 2 ps) and red lines representing the time average (every one ns). The times at which representative extended (E) and compact (C) conformers were extracted for display in Suppl. Fig. 10 are indicated. (**b**) Illustration of the distributions of the distances in (**a**) shown as histograms (red lines). The distributions show the existence of two distinct states for W_60_-Y_88_ (center) and W_76_-Y_88_ (right) indicating compact (C) and extended (E) conformations (see Suppl. Fig. 10). (**c**,**d**) Representative extended (**c**) and compact (**d**) conformations, respectively, showing relative positions of residues of p27-D2 involved in interaction with fragment hits.

To gain insight into the structural features of the small molecule binding sites within p27, we performed molecular dynamics (MD) simulations (over 400 ns) with p27-D2. The results recapitulated past MD findings (over 100 ns) for the longer p27-KID construct^21^ which revealed a transient β-hairpin involving residues W_60_-F_64_ and H_67_-L_70_ (within sub-region D2.1) and two α-helical turns involving residues E_80_-G_82_ and F_87_-Y_89_ (within sub-region D2.3). In the new MD trajectory, these secondary structures were stable on short nanosecond time-scales but unfolded and refolded over the longer time periods sampled in this experiment. These longer time-scale transitions were coupled with transient formation of hydrophobic clusters involving residues within sub-regions D2.1 and 2.2 (containing W_60_ and W_76_) and sub-region D2.3 (containing Y_88_; Fig. 9a) which gave rise to extended and compact conformations (labeled **E** and **C** in Fig. 9b). Representative extended and compact conformers are presented in Fig. 9c and d, respectively, and all others in Suppl. Fig. 10a,b. (Note that conformers are defined as compact if two of the three aromatic residues of p27 critical for binding to small molecules, W_60_, W_76_ and Y_88_, are less than 20 Å from each other.) We speculate that the extended and compact conformations create binding sites for Group 1 and Group 2 compounds, respectively. Our interpretation of these results is that Group 1 compounds bind to the F_87_YY_89_ motif within sub-region D2.3 in p27-D2 molecules in which Y_88_ is far away from W_60_ and W_76_ (>20 Å) and that Group 2 molecules bind to compact conformations when at least two of the three critical aromatic residues within the different sub-regions (sub-domains D2.1, D2.2 and D2.3) are clustered. Interestingly, analysis of the MD trajectory showed that Y_88_ and either W_60_ or W_76_ were frequently in close contact but that all three residues were rarely in close proximity (Suppl. Fig. 10c). This suggested that there are several different conformations with clustered aromatic residues (in particular, Y_88_ and either W_60_ or W_76_) capable of binding to Group 2 compounds, consistent with mutagenesis results showing that either W_60_ or W_76_, but not both, are dispensable for Group 2 compound binding (Suppl. Fig. 5a–f). In summary, the new MD results for p27-D2 suggest strongly that transient conformational fluctuations that create and disrupt clusters of aromatic residues modulate the binding of Group 1 and Group 2 small molecules to p27-D2. These results are consistent with the identification of W_60_, W_76_ and Y_88_ by NMR as the principal sites for compound binding and with results showing that binding is altered through mutation of these residues.

## Discussion

Drug discovery against folded proteins often involves identification of compounds that bind to sites that are naturally utilized for interactions with small molecule or macromolecular ligands. These types of binding sites are temporally stable and enable specific and tight interactions with chemically complementary small molecules. In contrast, disordered proteins (or disordered protein regions) exhibit dynamic and heterogeneous conformations that do not display similar, temporally stable small molecule binding sites. However, despite the lack of temporally stable feature, many disordered proteins/regions interact with macromolecular partners through the process of folding upon binding. We hypothesized that the ability of a disordered protein to bind other proteins would also confer the ability to bind small molecules and tested this idea through the studies of p27 described herein.

The entire p27 protein is disordered and its N-terminal domain (p27-KID) becomes ordered upon binding to Cdk2/cyclin A. A short linear motif within the D1 sub-domain of p27-KID (with the sequence R_30_NLFG_34;_ L, Leucine; Suppl. Fig. 11a,b) binds to a pocket on the surface of cyclin A. Sub-domain LH, which links sub-domains D1 and D2, contacts the surfaces of cyclin A and Cdk2 but contributes little to the binding energy^43^. Sub-domain D2 of p27 (p27-D2; 34 amino acids in length) adopts extensive secondary structure and makes extensive hydrophobic interactions with Cdk2 upon binding (Suppl. Fig. 11a,c and d). In addition, numerous hydrogen bonds form between residues within the D2 sub-domain and Cdk2. While p27-D2 exhibits 11 hydrophobic and aromatic residues (including I, L, V, F, W and Y residues), many of which contribute to interactions with Cdk2, this sub-domain does not independently form a stable hydrophobic core.

Despite extensive disorder in the unbound state, we identified two groups of small molecules that bound with exquisite specificity albeit with low affinity to two overlapping regions within p27. These molecules bound to regions containing aromatic rings, with preference for W and Y residues. Molecules in Group 1 bound to a localized region, F_87_YY_89_, while molecules in Group 2 bound to this region as well as two others containing two W residues (W_60_ and W_76_). Strikingly, molecules within Groups 1 (25 molecules) and 2 (14 molecules), respectively, were chemically similar, demonstrating chemical structure-binding activity relationships for these two types of small molecule: protein interactions. We refer to this phenomenon as “fuzzy structure activity relationships (SAR)” due to the “fuzzy” character of the respective interaction potential maps (Fig. 3a,b).

These chemical features of the p27 binding molecules can be rationalized based upon the features of residues within and flanking the NMR-identified binding sites within p27-D2. Group 1 and 2 molecules exhibited two or three heterocyclic aromatic rings, and exhibited the potential for these rings to participate in hydrophobic interactions (Fig. 3a,b; gold polygons). Molecules in both groups also displayed the potential to bind electropositive moieties (Fig. 3a,b; cyan polygons), consistent with the F_87_YY_89_ region in the Group 1/2 binding sites being flanked on the C-terminal end by R_90_PPR_93_, and W_60_ in the Group 2 binding site being flanked on the N-terminal end by R_58_K_59_. Residue W_76_ within the Group 2 binding site is flanked by amino acids with both electronegative and electropositive, as well as polar, features (E_71_GKYEW_76_QEVEK_81_) although the potential for interactions with electronegative moieties was only weakly represented (Fig. 3b; red polygons). In addition to electrostatic features, the small molecules, which exhibit hydrogen bond donors and acceptors, may achieve specificity through transient hydrogen bonds with complementary groups within p27-D2, as observed for small molecules binding to Myc^44^. We argue that these and other currently unappreciated features of the p27-D2 polypeptide chain create the potential to specifically bind Group 1 and 2 small molecules.

What are the conformational features of small molecule binding sites within p27? Our MD computations with p27-D2 revealed dynamic fluctuations of pairwise distances between the aromatic residues (specifically, W_60_, W_76_ and Y_88_) within the Group 2 binding sites (Fig. 9). While W_60_ and W_76_ sampled a relatively narrow distance range (16.5 ± 2.3 Å), the distances between W_60_ and Y_88_, and W_76_ and Y_88_, fluctuated over a wider range (20.5 ± 5.5 Å and 19.6 ± 4 Å, respectively). Furthermore, these latter residue pair distances each exhibited two discrete populations that we term compact (**C**) and extended (**E**; Fig. 9b, middle and right panels). We propose that the compact conformations create binding sites for Group 2 compounds, consistent with NMR CSP patterns (Fig. 7c). In addition, we propose that extended conformations favor the binding of Group 1 compounds to F_87_YY_89_ region, also consistent with NMR CSP patterns (Fig. 7b). W_60_ is flanked by F_62_ and F_64_, and W_76_ by Y_74_, but mutagenesis results (Suppl. Fig. 5a–f) suggested that the W residues are the principal determinants of Group 2 compound binding. This is probably due to the potential of side chains of W and Y residues (but not of F residues) to form hydrogen bonds with small molecules, in addition to participating in hydrophobic and π-stacking interactions. Interestingly, distance correlation analysis (Suppl. Fig. 10c) indicated that, in the compact conformations, Y_88_ is most frequently close to either W_60_ or W_76_ but rarely close to both W residues. This suggested that close proximity of Y_88_ and one or the other of the two W residues created binding sites within p27-D2 for Group 2 compounds. This observation is also consistent with mutagenesis results, which showed that mutation of both W residues, but not of individual W residues, abrogated binding to Group 2 compounds (Suppl. Fig. 5c, f *versus* a, b, d, e). In summary, p27-D2 exhibited transient close contacts between W_60_ or W_76_ and Y_88_, which we propose creates binding sites for Group 2 compounds. Additionally, when neither W residue and Y_88_ are close, the spatial proximity of the three aromatic residues in the F_87_YY_89_ region created binding sites for Group 1 compounds. It is interesting that, in the Cdk2/cyclin A-bound structure of p27-KID^34^, the five aromatic residues within sub-regions D2.1 and D2.2 (that bind to Group 2 compounds) are in close proximity (but separated from the F_87_YY_89_ region; Suppl. Fig. 11a,c and d). The MD results show that, in the absence of Cdk2/cyclin A, subsets of these eight aromatic residues transiently interact, sometimes creating binding sites for different types of heterocyclic aromatic small molecules (Group 1 or 2).

The compound, SJ403, was demonstrated to sequester p27-D2 away from Cdk2/cyclin A and activate Cdk2 catalytic activity, effectively fulfilling our goal of inhibiting p27’s cell cycle inhibitory function. While the affinity of Group 1 and 2 molecules is low, they do exhibit high specificity for particular regions of p27. As discussed above, residues within these regions otherwise engage Cdk2. Remarkably, the >2,300 compounds that were screened failed to bind other regions of p27 (sub-domains D1 and LH), suggesting that these other regions lack a sufficient density of aromatic residues (specifically, W and Y residues) to specifically recognize small heterocyclic aromatic molecules. Sub-domain LH, in isolation, does not bind to Cdk2/cyclin A but sub-domain D1 binds cyclin A with high affinity (K_d_, 42 nM)^45^. We speculate that the RxLFG motif within this latter region, due to its limited length, cannot adopt conformations that create binding pockets for small molecules, as is possible for the much longer D2 sub-domain. However, the low affinity of the Group 1 and 2 compounds for p27 limits their applicability toward our broader goal of modulating p27 function in cells and, ultimately, humans. How can the affinity of small molecules for p27 be increased? We propose that the Group 1 and 2 molecules cause a degree of conformational restriction within p27-D2 and that molecules that enhance this restriction will exhibit higher affinity. We envision that small molecules with greater “three-dimensionality”, that present chemically diverse and complex features, will be better templates for binding and sequestering p27. Efforts based on two strategies are underway to optimize our fragment hits using synthetic chemistry. First, we are “growing” the Group 1 and 2 scaffolds by introducing diverse chemical moieties at various positions on the heterocyclic ring systems to enable additional interactions with residues near W_60_, W_76_, and Y_88_ within p27-D2. Second, when the growing experiments are complete, we will synthetically “link” the optimal Group 1 and Group 2 molecules to further enhance binding to p27-D2. The results of these future experiments will indicate whether synthetic strategies for compound optimization that have emerged from structure-based drug discovery can be applied to a disordered protein. In conclusion, we have discovered small molecules with “fuzzy SAR” that mediate specific binding to and inhibition of p27, demonstrating the potential to rationally “drug” disordered protein targets in the future.

## Methods

### Preparation of proteins

The p27 constructs were expressed in *E. Coli* with an N-terminal 6xHis affinity tag after sub-cloning into pET28a (Novagen) using established procedures^20^. This included p27-KID (residues 22-105 of human p27) and the following mutants: W_60_A, W_60_F, W_76_A, W_76_F, W_60_A-W_76_A, W_60_F-W_76_F, F_87_A, Y_88_A, Y_89_A, and R_90_A. p27-D2 (residues 58-105 of human p27) and the mutant, C_99_S-R_93_C, were expressed similarly. Isotope-labeled proteins (^15^N, ^13^C/^15^N, and ^2^H/^13^C/^15^N) were expressed in a MOPS-based minimal media using established procedures^22^. All p27 constructs were purified by nickel affinity chromatography, digested with thrombin to remove the 6xHis tag, and further purified using reverse-phase high performance liquid chromatography (HPLC) using a C4 column (Vydac) and 0.1% trifluoroacetic acid-containing water/acetonitrile solvent system. Protein concentrations were determined by UV absorbance at 280 nm using a molar extinction coefficient of 15,470 M^−1^ cm^−1^ for p27-KID, p27-KID-F_87_A, p27-KID-R_90_A, p27-D2, and p27-D2-C_99_S-R_93_C; 13980 M^−1^cm^−1^ for p27-KID-Y_88_A and p27-KID-Y_89_A; 9,970 M^−1^ cm^−1^ for p27-KID variants with a single tryptophan residue; and 4,470 M^−1^ cm^−1^ for p27-KID variants without a tryptophan residue. Full length human Cdk2, active Cdk2 (phosphorylated at threonine 160), truncated human cyclin A (residues 173–432), and p21-KID were expressed and purified using established protocols^20^,^46^.

### Preparation of ^2^H/^13^C/^15^N-p27-D2/Cdk2/Cyclin A ternary complex

The Cdk2/cyclin A complex was prepared by equilibrating Cdk2 and cyclin A (1:1 mole ratio) for 1 hour at 4 °C followed by purification using size exclusion chromatography (S75 resin, GE Healthcare, Piscataway, NJ) in buffer containing 20 mM Tris, pH 7.5, 200 mM NaCl, 5 mM TCEP). The ternary complex was prepared by equilibrating ^2^H/^13^C/^15^N-p27-D2 with Cdk2/cyclin A(1.1:1 mole ratio) at 4 °C overnight followed by purification using size exclusion chromatography (S200 resin, GE Healthcare, Piscataway, NJ) in the same buffer as the Cdk2/cyclin A complex. For NMR experiments, the ^2^H/^13^C/^15^N-p27-D2/Cdk2/cyclin A complex was buffer exchanged into 20 mM Na phosphate, pH 6.5, 200 mM NaCl, 10 mM DTT-D_10_ and 10% ^2^H_2_O.

### Fragment libraries

The fragment library used to screen for binding to p27-KID was composed of two collections that were designed according to different criteria: (a) 1,100 fragments were purchased from the Maybridge Ro3 Diversity Fragment Library (‘**Maybridge**’)^47^, and (b) 1,222 fragments were purchased from Enamine (N = 250) and Life Chemicals (N = 972) using an in-house algorithm (‘**In-House**’).

The **Maybridge** fragment collection was designed to provide broad coverage of chemical space for fragment-based drug discovery. Each fragment satisfies Congreve’s Rule of Three^40^: (a) molecular weight < 300; (b) number of hydrogen bond donors ≤ 3; (c) number of hydrogen bond acceptors ≤ 3; and (d) clogP ≤ 3. All compounds have experimentally determined equilibrium solubility ≥200 mM (DMSO) and ≥1 mM (PBS) and confirmed purity ≥95% (based upon analysis by liquid chromatography/mass spectrometry), and are free of reactive or toxic functional groups. The 1,100 fragments constitute a ‘core’ set that encompasses the chemical diversity of the entire collection (1,823 fragments).

The **In-House** fragment collection consists of 1,222 commercially-available compounds selected using a custom algorithm designed to identify structurally complex, low molecular weight molecules with scaffolds that were well-sampled within the separate St. Jude high-throughput screening library (HTS library, >500,000 compounds; see below). First, commercial fragment collections (subsets of larger diversity collections filtered for ‘fragment-like’ characteristics) were filtered to remove molecules containing inorganic atoms, isotopes, or invalid structures, and to remove molecules that were not available in sufficient quantity (<50 mg). Passing molecules were abstracted to Murcko scaffolds using Pipeline Pilot (‘Generate Fragments’ component in Accelrys v. 8.5 with alpha atoms preserved, see ref. 48 for the general method). These scaffolds were further filtered according to the following rules: number of reactive substructures = 0 (‘REOS’ filters^49^,^50^,^51^, number of rotatable bonds <= 3, number of heavy atoms >= 10, number of rings >= 1 and number of ring substitutions >1 for single ring systems, and number of molecules present in the St. Jude HTS library containing the scaffold >= 8. Molecules containing these scaffolds were identified in the commercial fragment libraries, and then prioritized for purchase according to highest Oprea complexity^52^. This library has the following average calculated physicochemical properties: MW = 246 ± 39 Da, number of atoms = 17 ± 3, log P = 1.7 ± 1.0, polar surface area = 63 ± 19 Å^2^, number of H-bond acceptors = 4.3 ± 1.4, and number of H-bond donors = 1.3 ± 0.9. The distributions of these and other chemical features of the two fragment libraries are summarized in Suppl. Fig. 12a.

### Field alignment analysis of molecules that bound to p27-KID

Consensus field maps for Group 1 and 2 molecules were defined using the FieldTemplater module in Forge (v10, Cresset, REF= http://www.cresset-group.com/products/forge/accessed Dec 2014). The FieldTemplater module takes as input a set of reference molecules (the active compounds for Groups 1 and 2 identified in the first two rounds of screening; Suppl. Table 1A, B) and seeks to align them in order to maximize overlap between their interaction fields. Reference molecules are first aligned using field points, followed by slower, more accurate optimization of the alignment using the full interaction field. Prior to alignment, conformations were generated using the ‘Very Accurate and Slow’ conformation hunt option in Forge and default settings. The similarity scoring function used in the alignment was based on 100% field similarity and 0% shape similarity in order to maximize the topological diversity of molecules retrieved using the model in the next round of screening.

The consensus field maps for Group 1 and 2 molecules were used to query a database of 10,455 fragment-like molecules from ChemDiv (www.chemdiv.com) using the FIeldScreen module in Forge. Of the 215 molecules with the highest field similarity scores, 184 compounds were purchased. However, 106 of these were poorly soluble under our assay conditions and not screened. From the remaining 78 compounds, 12 additional hits were identified using 2D ^1^H-^15^N HSQC NMR (see below).

### NMR experiments

Screening and validation NMR experiments were performed at 298 K (25 °C) using either a Varian Inova 600 MHz spectrometer equipped with a triple resonance (HCN) room temperature gradient probe or a Bruker Avance 600 MHz spectrometer equipped with TCI cryogenic gradient probe and a SampleJet sample changer. Fragment molecules were initially analyzed as pools of five molecules dissolved at 10 mM each in DMSO-D_6_. The fragment pools contained in 96-well plates were mixed using a Gilson 215 liquid handler with buffer (20 mM Na phosphate, pH 6.5, 20 mM NaCl, 10% ^2^H_2_O, 5 mM DTT-D_10_) or buffer containing the p27-KID protein (10 μM p27-KID) to give final compound concentrations of 200 μM each. For initial fragment screening experiments, one-dimensional (1D) ^1^H- and WaterLOGSY^35^ NMR spectra were recorded for compound pools without and with protein. Pools exhibiting hits were deconvoluted by analyzing pure compounds using 1D ^1^H and validated by two-dimensional (2D) heteronuclear NMR experiments (2D ^1^H-^15^N HSQC titrations) using the Bruker Avance 600 MHz spectrometer. NMR samples used for 2D ^1^H-^15^N HSQC titrations contained 100 μM ^15^N-labeled p27 protein (p27-KID, p27-KID mutants, or p27-D2) in 20 mM Na phosphate, pH 6.5, 200 mM NaCl, 10% ^2^H_2_O, 5 mM DTT-D_10_; compounds dissolved in DMSO-D_6_ were titrated to the desired concentrations. DMSO-D_6_ was added to maintain a constant concentration (2% vol/vol). A spectral resolution of 3.5 and 5.7 Hz was achieved in the ^1^H and ^15^N dimensions, respectively. Tryptophan and tyrosine, respectively, were titrated into ^15^N-p27-KID (100 μM) up to 3 mM. Three-dimensional (3D) backbone triple-resonance experiments to establish resonance assignments for the p27 constructs were performed using the Bruker Avance 600 MHz spectrometer. Assignments for p27-KID and p27-D2 are illustrated in Suppl. Fig. 12b,c and d,e, respectively. 2D ^1^H-^15^N TROSY-HSQC NMR experiments with ^2^H/^13^C/^15^N-p27-D2/Cdk2/cyclin A and SJ403 have been recorded at 308 K (35 °C). 2D ^1^H-^15^N HSQC experiments of representative fragment hits (1 mM of SJ319843, group 1 and SJ403, group 2, respectively) with ^15^N-p21-KID (20 μM) were recorded at 298 K using a Bruker Avance 600 MHz spectrometer. NMR spectra were processed using Bruker Topspin software and analyzed using computer-aided resonance assignment (CARA) software^53^.

### Analysis of 2D ^1^H-^15^N HSQC titrations

Chemical shift perturbations are generally quantified as combined ^1^H and ^15^N chemical shift values. However, the analysis of primary data for small molecules binding to p27 showed that the largest CSPs were for the ^1^H dimension, which were statistically significant, and that the corresponding ^15^N CSP values were often small and not significant. Thus, the use of a combined chemical shift values would mask the effects of compound binding. We also rigorously considered the magnitude of CSPs relative to the experimental digital resolution of the ^1^H and ^15^N dimensions of HSQC spectra, and a threshold defined as the average CSP value plus two times the standard deviation of the mean (Δδ_ave_ + 2σ). Thus, the assessment of statistical significance was based upon whether a particular CSP value was larger than, i) the experimental spectral resolution in the given dimension (^1^H or ^15^N) and ii) the quantity, Δδ_ave_ + 2σ, for that dimension.

### Determination of binding affinity for p27:small molecule interactions by NMR

Sixteen point titrations of representative fragment hits from Group 1 and Group 2 (SJ572710 and SJ572403, respectively) into ^15^N-p27-KID (100 μM) were recorded using an adopted “in-phase” 2D ^1^H-^15^N HISQC with proton decoupling during ^15^N chemical shift labeling achieved with a WALTZ16 composite pulse with an amplitude of 7.1 kHz^54^,^55^. All experiments were collected at a Bruker Avance 800 MHz spectrometer equipped with TCI cryogenic gradient probe. The following molar ratios of ^15^N-p27-KID (100 μM) to inhibitor were used: 1:0, 1:0.1, 1:1.5, 1:3, 1:4.5, 1:6, 1:7.5, 1:9, 1:10.5, 1:12, 1:15, 1:18, 1:21, 1:24, 1:27 and 1:30. Each spectrum was recorded with 256 (t_1,max_ = 31.0 ms) and 1024 (t_2,max_ = 106.5 ms) complex points in the ^15^N and ^1^H dimensions, respectively, with eight transients collected per point. The spectral resolution of the ^1^H and ^15^N dimensions was 2.4 and 1.7 Hz, respectively. Chemical shift perturbations throughout the entire titration had to be greater than this resolution threshold to be considered for further analysis. The data was processed using the NMRPipe package^56^ and analyzed using in-house scripts written in Python using the Scipy computing libraries and Mathematica (Wolfram Research). In order to alleviate human bias in the peak position determination the automatic peak picking function in NMRpipe was utilized in which a spectral window was assigned for each resonance over the entire trajectory of the titration for a given chemical shift. The error in the peak position for a given resonance was taken as the ^15^N or ^1^H^N^ line width divided by the signal-to-noise ratio. All resonances that exhibited chemical shift perturbations greater than the spectral resolutions and Δδ_ave_ + 2σ were subsequently grouped and fitted globally for their respective maximum chemical shift difference and a global dissociation constant (K_d_). The error in the K_d_ values was determined using a Monte-Carlo approach in which an error of 10% was imposed on the ligand concentration and the error in the chemical shift’s peak position was considered.

### Fluorescence anisotropy experiments

The p27-D2-C_99_S-R_93_C mutant was conjugated with Alexa Fluor488-C_5_-Maleimide (Life Technologies, p27-D2-FL) in buffer containing 20 mM Na phosphate, pH7.3, 20 mM NaCl according to the manufacturer’s protocol. The conjugated protein was further purified by reverse-phase high performance liquid chromatography (HPLC) using a C4 column (Vydac) and 0.1% trifluoroacetic acid-containing water/acetonitrile solvent system. Lyophilized HPLC fractions were resuspended in buffer containing 20 mM Na phosphate, pH 7, 200 mM NaCl, 3.5 mM TCEP. Fluorescence anisotropy measurements were performed at 25 °C on a Horiba Fluorolog 3 spectrofluorometer. Briefly, p27-D2-FL (20 nM) was mixed with Cdk2/Cyclin A (300 nM) and added to the required amount of lyophilized compound (SJ403). All samples have been incubated overnight in the dark at 4 °C prior to fluorescence measurements. Fluorescence anisotropy binding data was analyzed using KaleidaGraph. Curve fitting was performed on the average of three independent experiments by a non-linear regression binding model.

### Cdk2/cyclin A kinase activity assays

Cdk2/Cyclin A (200 pM) was mixed with Histone H1 (15 μM; EMD Millipore), p27-D2 (200 nM) and varied amounts of lyophilized SJ403 and incubated overnight at 4 °C. The effect of the SJ403 compound on Cdk2 activity was determined by performing similar experiments in the absence of p27-D2. Subsequently, ATP (50 μM total concentration, of which 6 μCi γ ^32^P-ATP (PerkinElmer, Inc)) was added to each reaction and further incubated for 35 minutes at 30 °C. Each reaction had a total volume of 20 μL. The sample buffer contained 20 mM HEPES pH 7.3, 25 mM sodium β-glycerolphosphate, 15 mM MgCl_2_, 16 mM EGTA, 0.5 mM Na_3_VO_4_ and 10 mM DTT. The reactions were quenched by addition of SDS-gel loading buffer (5 μL) and then analyzed by SDS-PAGE (10 μL). The gels were dried at 70 °C under vacuum and a phosphoimager (GE Healthcare, Piscataway, NJ) was used to quantify the ^32^P-Histone H1 bands. IC_50_ values were determined by curve fitting using KaleidaGraph software. Experiments were performed in triplicate and mean IC_50_ and standard deviations of the mean values are reported.

### Molecular dynamics simulation experiments

All-atom MD simulations using graphics processing unit (GPU)-optimized AMBER 12 software^57^ were used to explore the conformational landscape of the p27-D2 domain. The conformation of p27-D2 within the p27-KID/Cdk2/cyclin A structure (PDB ID, 1JSU) was used as the starting structure from MD computations with amino acid protonation states modified to reflect pH 7.0. The structure was placed in a rectangular box of TIP3P water that was 15 Å larger on all sides than the p27-D2 molecule. In addition to neutralizing the system by adding counter ions, 20 mM NaCl was added to mimic the experimental conditions in our simulations.

The system was equilibrated and stabilized using multi-step energy minimization at 298 K, as described previously^58^. All production runs were performed using the constant number of particles, volume and energy (NVE) ensemble with periodic boundary conditions. The particle-mesh Ewald (PME) method was used for electrostatic interactions and a 10 Å cut-off was used for Lennard-Jones interactions. The SHAKE algorithm was used to restrict the motions of all covalently bonded hydrogen atoms. Simulations were performed at 298 K and 1 atm pressure. The total time-scale for our simulation was 0.4 microseconds with snapshots being stored every 2 ps, resulting in a total of 200,000 snapshots from the trajectory.

## Additional Information

**How to cite this article**: Iconaru, L. I. *et al.* Discovery of Small Molecules that Inhibit the Disordered Protein, p27^Kip1^. *Sci. Rep.* **5**, 15686; doi: 10.1038/srep15686 (2015).

## Supplementary Material

Supplementary Information
