# Supplementary material for: Discovery of Small Molecules that Inhibit the Disordered Protein, p27Kip1
Source: Sci Rep. 2015 Oct 28;5:15686. doi: 10.1038/srep15686 (PMC4623604; doi:10.1038/srep15686)
Supplement: Supplementary Information [file srep15686-s1.pdf]

Discovery of Small Molecules that Inhibit the Disordered Protein, $p27^{Kip1}$

Luigi I. Iconaru[1,2], David Ban[1], Kavitha Bharatham[3,4], Arvind Ramanathan[5], Weixing Zhang[1], Anang A. Shelat[3], Jian Zuo[2#] and Richard W. Kriwacki [1,6#]

[1] Department of Structural Biology; [2] Department of Developmental Neurobiology; [3] Department of Chemical Biology and Therapeutics, St. Jude Children's Research Hospital, Memphis, TN 38105
[4] Present address: Center for Chemical Biology and Therapeutics, Institute for Stem Cell Biology and Regenerative Medicine, GKVK, Bellary Road, Bangalore 560065, India
[5] Computational Science and Engineering Division, Health Data Sciences Institute, Oak Ridge National Laboratory, Oak Ridge, TN 37830
[6] Department of Microbiology, Immunology and Biochemistry, The University of Tennessee Health Science Center, Memphis, TN 38163

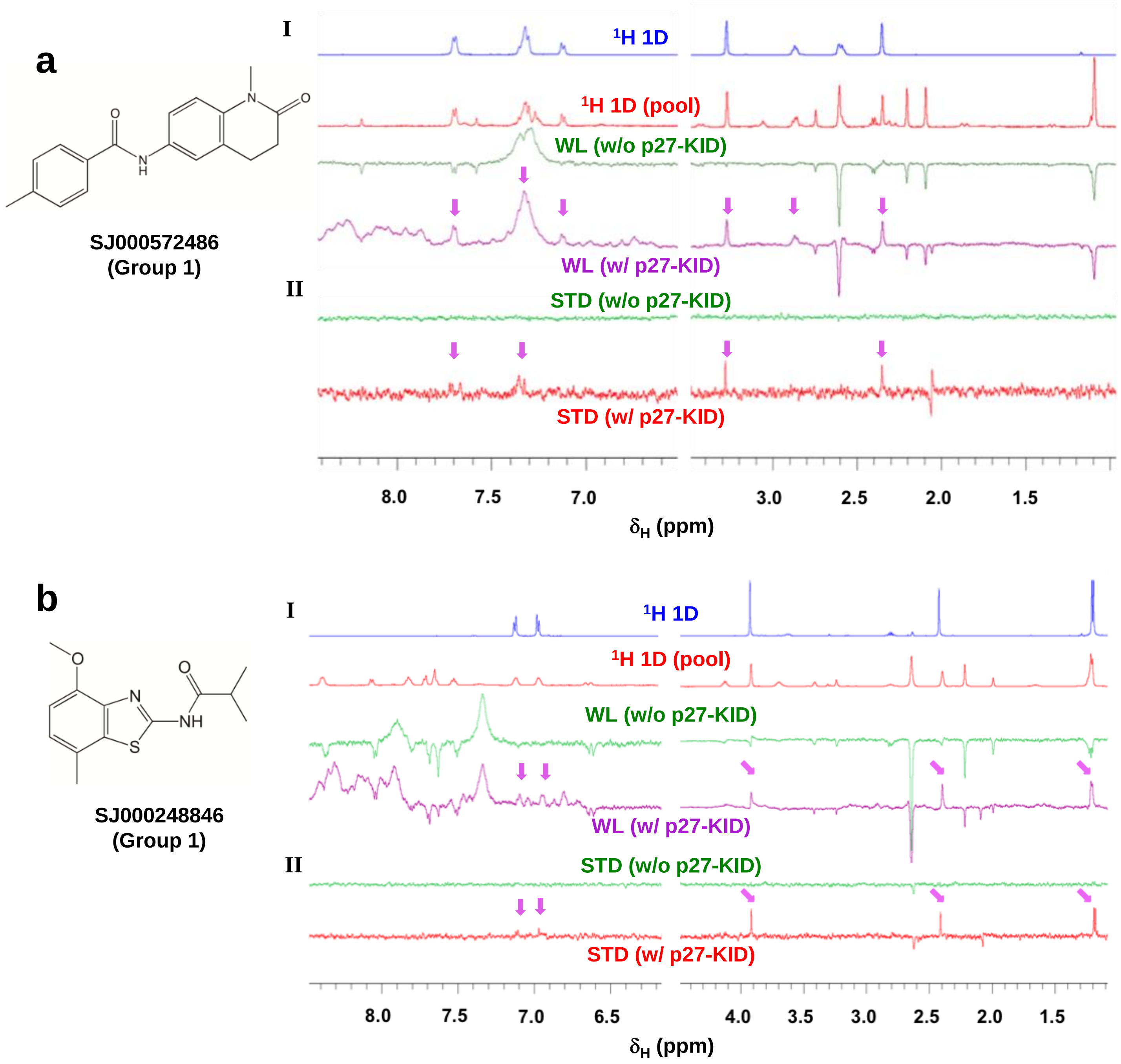

Supplementary Figure 1. Representative 1D NMR results showing that small molecules bind to p27-KID. **(a, b)** Representative 1D NMR results for two Group 1 molecules binding to p27-KID. 1D ^{1}H spectra are shown for the "hit" molecules and for the pool of five compounds (pool) in which they were originally screened. Also illustrated are WaterLOGSY (WL) and saturation transfer difference (STD) spectra for the compounds in the presence (w/) and absence (w/o) of p27-KID. The chemical structures of the two compounds are illustrated at the left.

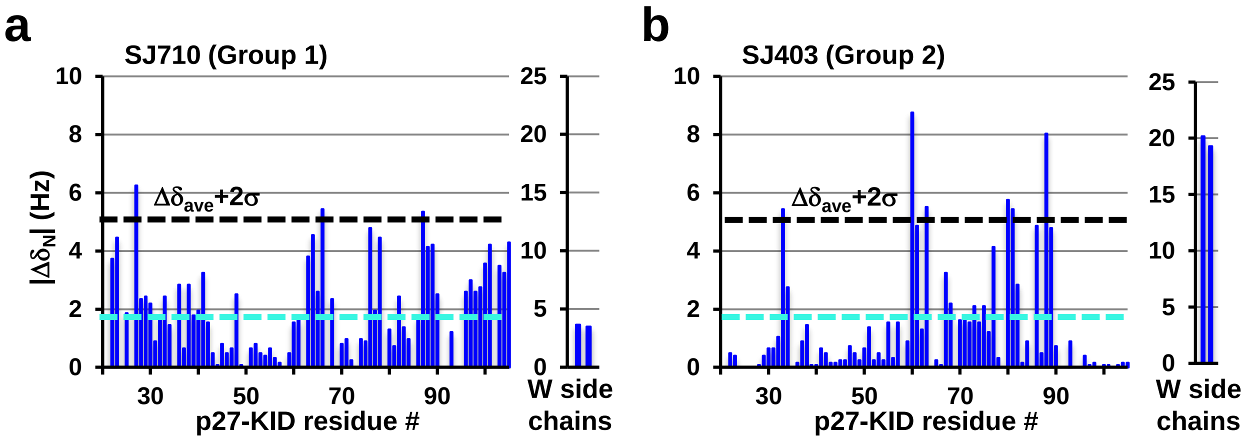

Supplementary Figure 2. Individual ^{15}N ($\Delta\delta_N$) chemical shift perturbations obtained through analysis of 2D ^{1}H-^{15}N "in-phase" HSQC NMR titrations of SJ710 (**b,** Group 1) and SJ403 (**c**, Group 2), respectively, into ^{15}N-p27-KID (100 μM).

a

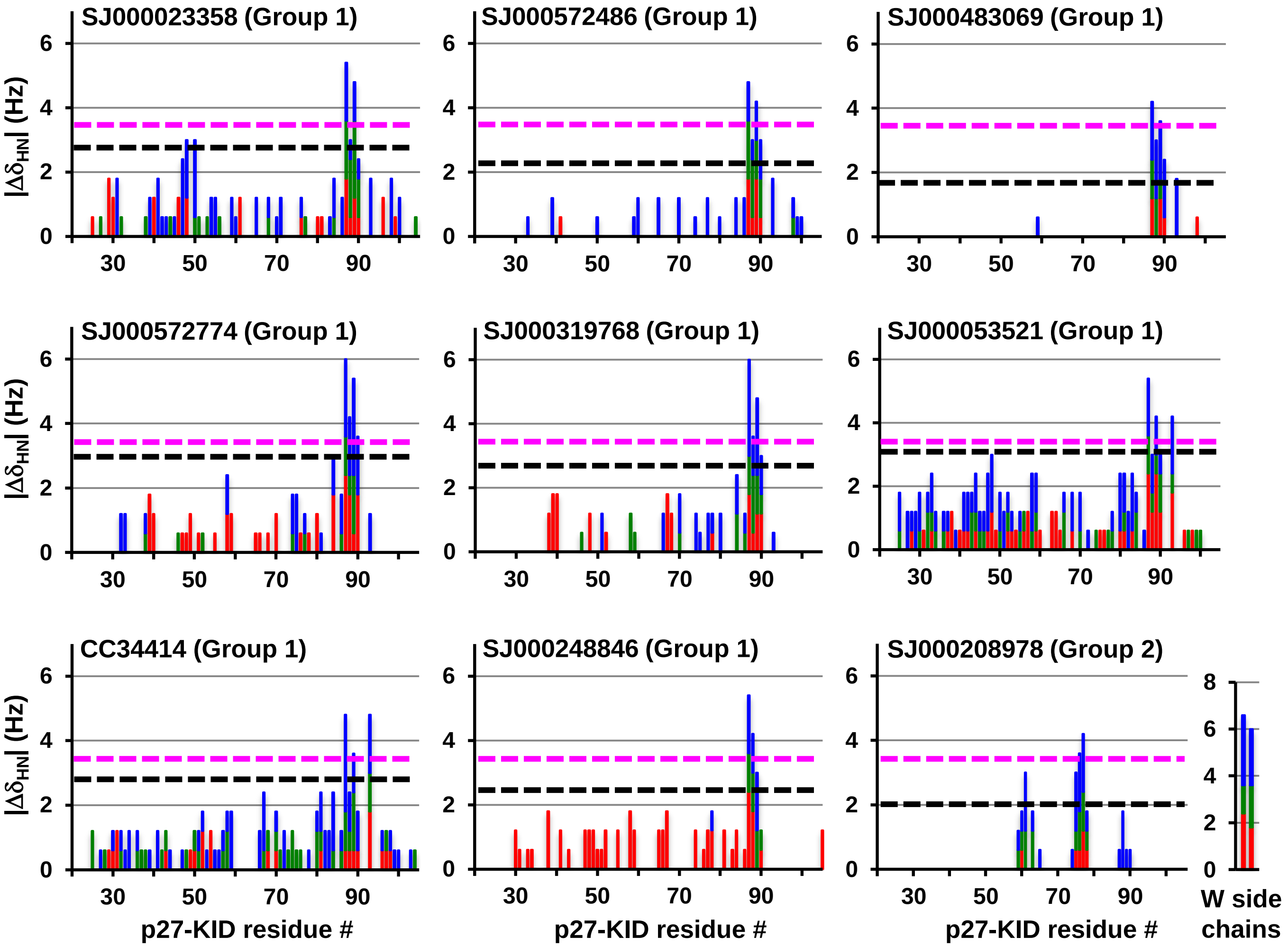

Supplementary Figure 3. Results of 2D ^{1}H-^{15}N HSQC analysis of all small molecules that exhibited binding to ^{15}N-p27-KID. Individual ^{1}H chemical shift perturbation histograms are shown for the molecules identified as "hits" in the initial fragment library screen (**a**), in the follow-up screen of chemically similar compounds in the same libraries (**b**), and from the in-house high-throughput screening library identified on the basis of the field alignment models (**c**). The threshold for identifying specific interactions with p27-KID residues was defined as two standard deviations above the average the perturbation values (represented by a dotted black line in each graph). The experimental spectral resolution in the ^{1}H dimension (3.5 Hz) is represented by the dotted magenta line. The molar ratios of ^{15}N-p27-KID (100 μM) to inhibitor used for titrations were 1:2.5 (red), 1:5 (green), and 1:10 (blue).

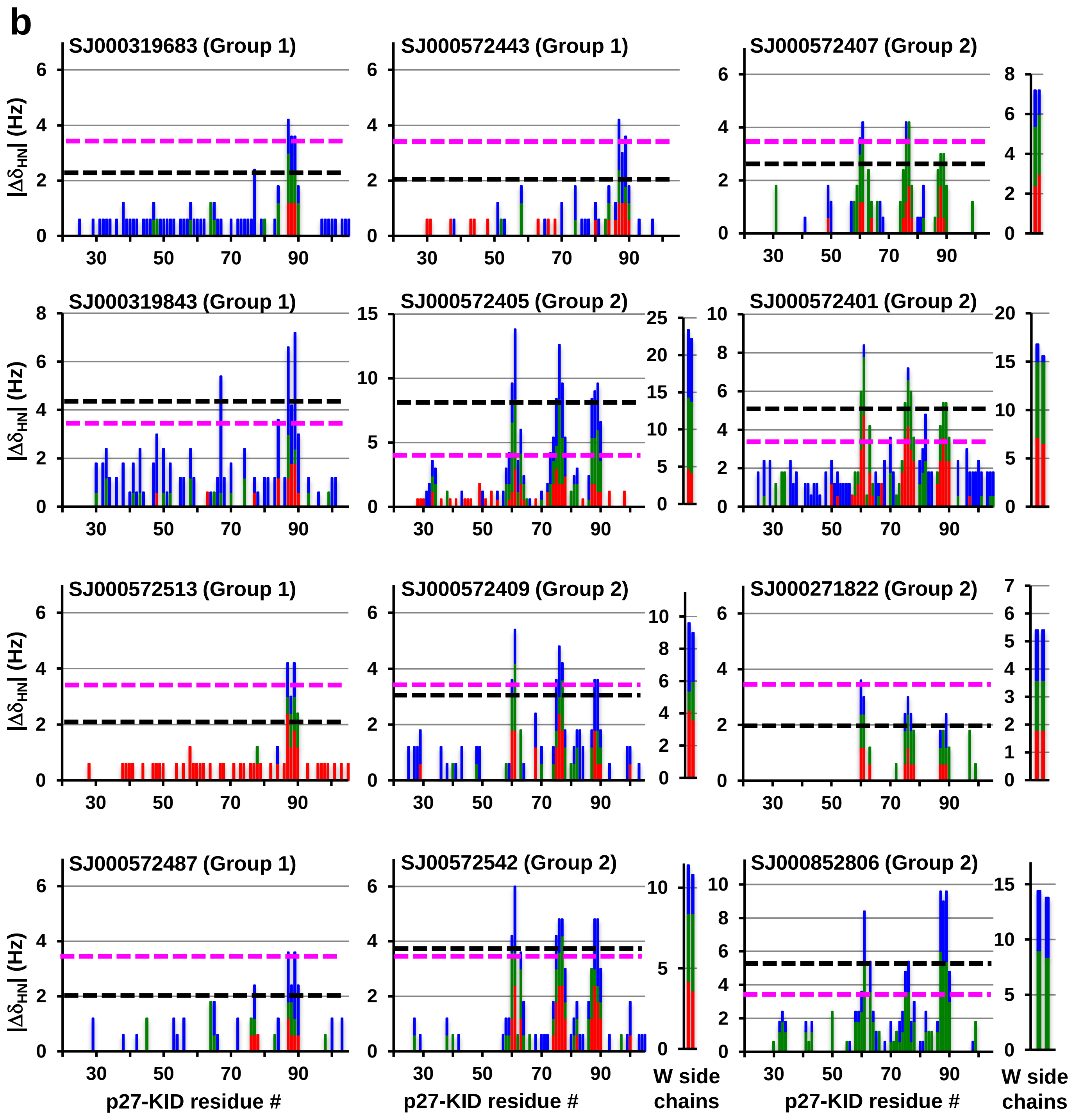

Supplementary Figure 3 (continued)

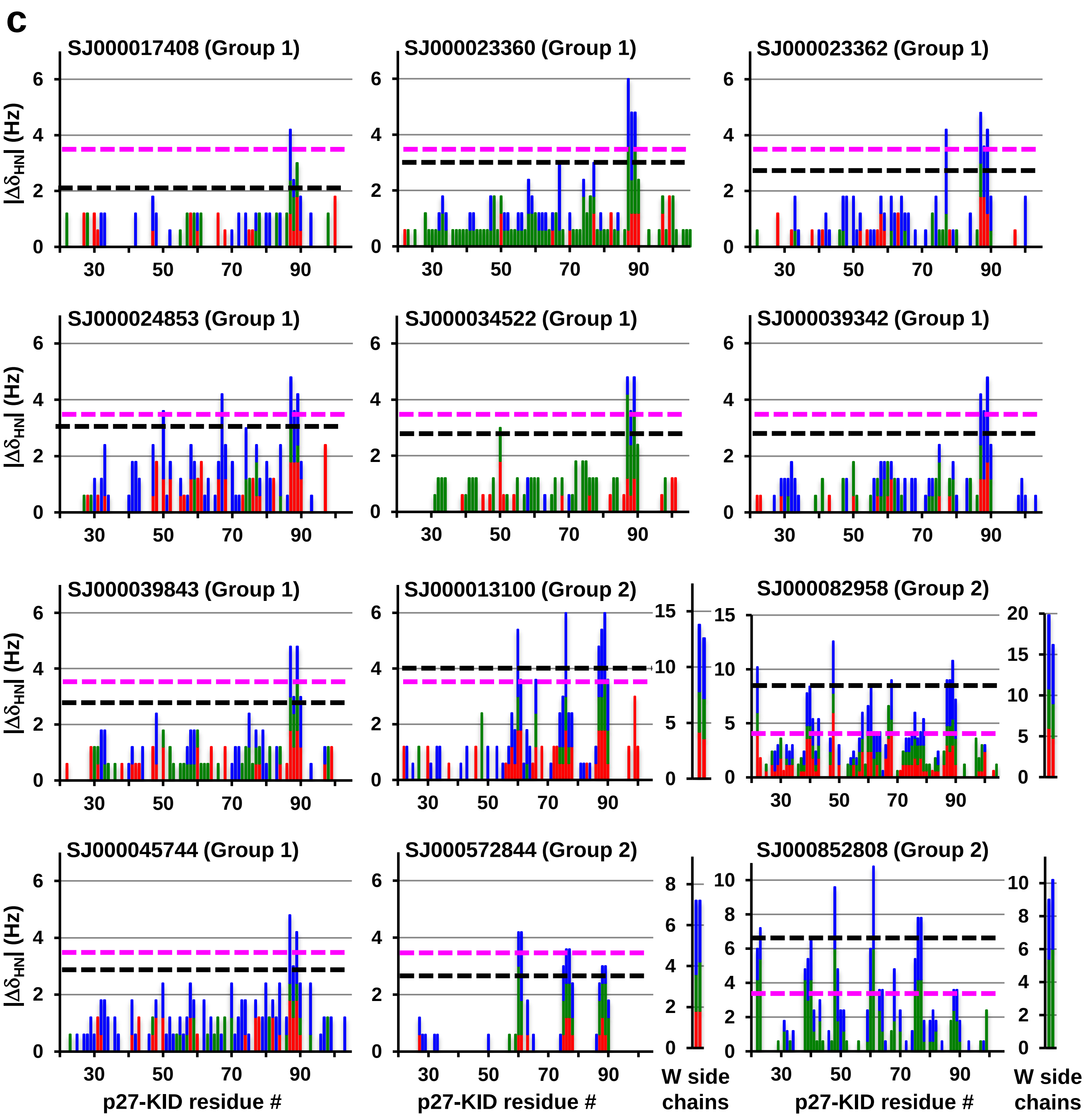

Supplementary Figure 3 (continued)

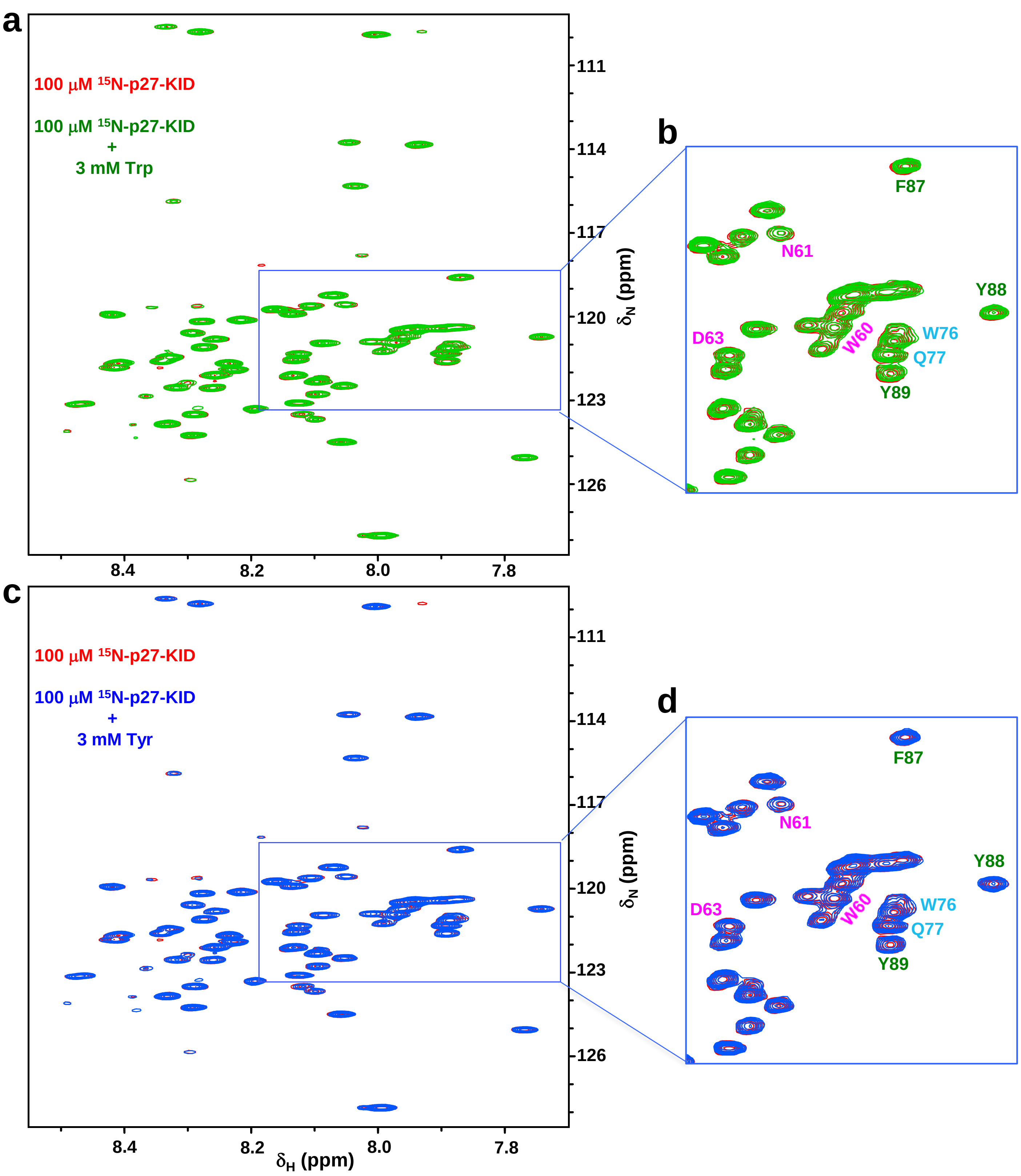

Supplementary Figure 4. Amino acids with aromatic side chains do not bind specifically to p27. (**a, c**) Overlaid 2D ^{1}H-^{15}N HSQC NMR spectra of ^{15}N-p27-KID (100 µM) in the absence (red) and presence of (**a**) tryptophan (3 mM, green) and (**c**) tyrosine (3 mM, blue)**.** (**b, d**) Expanded regions (represented by the blue boxes) including a subset of the p27-KID residues involved in interaction with fragment hits (highlighted in Fig. 2b, e) show no perturbations after addition of tryptophan (**b**) and tyrosine (**d**), respectively.

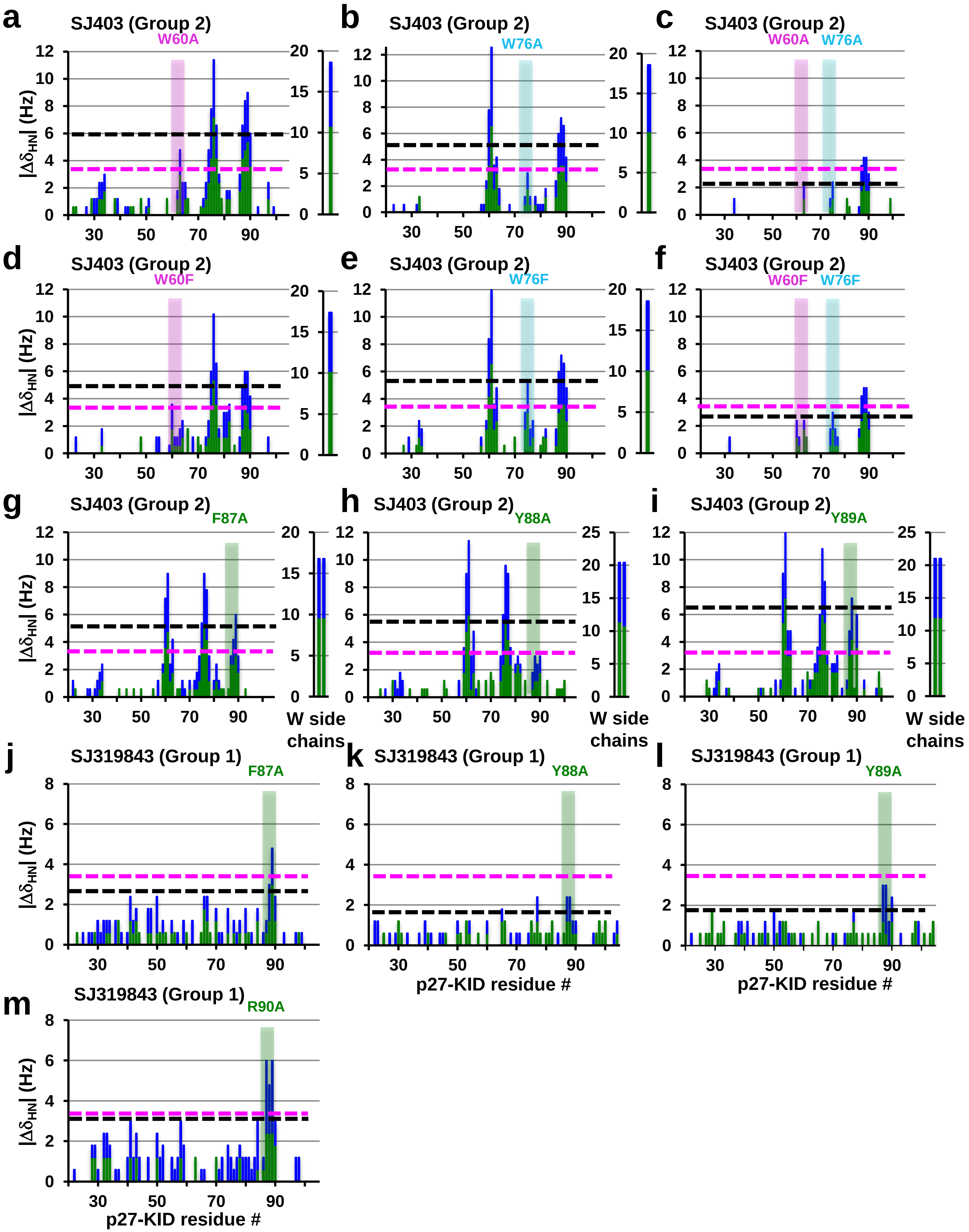
a
SJ403 (Group 2)
W60A
b
SJ403 (Group 2)
W76A
c
SJ403 (Group 2)
W60A W76A
d
SJ403 (Group 2)
W60F
e
SJ403 (Group 2)
W76F
f
SJ403 (Group 2)
W60F W76F
g
SJ403 (Group 2)
F87A
h
SJ403 (Group 2)
Y88A
i
SJ403 (Group 2)
Y89A
W side chains
j
SJ319843 (Group 1)
F87A
k
SJ319843 (Group 1)
Y88A
l
SJ319843 (Group 1)
Y89A
m
SJ319843 (Group 1)
R90A
$|\Delta\delta_{HN}|$ (Hz)
p27-KID residue #

Supplementary Figure 5. Results of 2D NMR analysis of mutant forms of p27-KID to determine the contributions of tryptophan and tyrosine residues to interactions with small molecules. Histograms of ^{1}H chemical shift perturbations observed in 2D ^{1}H-^{15}N HSQC spectra with increasing concentrations of SJ403 for, (**a**) ^{15}N-p27-KID-$W_{60}A$, (**b**) ^{15}N-p27-KID-$W_{76}A$, (**c**) ^{15}N-p27-KID-$W_{60}A$-W76A, (**d**) ^{15}N-p27-KID-$W_{60}F$, (**e**) ^{15}N-p27-KID-$W_{76}F$, (**f**) ^{15}N-p27-KID-$W_{60}F$-$W_{76}F$, (**g**) ^{15}N-p27-KID-$F_{87}A$, (**h**) ^{15}N-p27-KID-$Y_{88}A$, (**i**) ^{15}N-p27-KID-$Y_{89}A$; and of SJ319843 for (**j**) ^{15}N-p27-KID-$F_{87}A$, (**k**) ^{15}N-p27-KID-$Y_{88}A$, (**l**) ^{15}N-p27-KID-$Y_{89}A$, and (**m**) ^{15}N-p27-KID-$R_{90}A$. Ratios of ^{15}N-p27-KID to small molecules of 1:5 (green) and 1:10 (blue) were used.

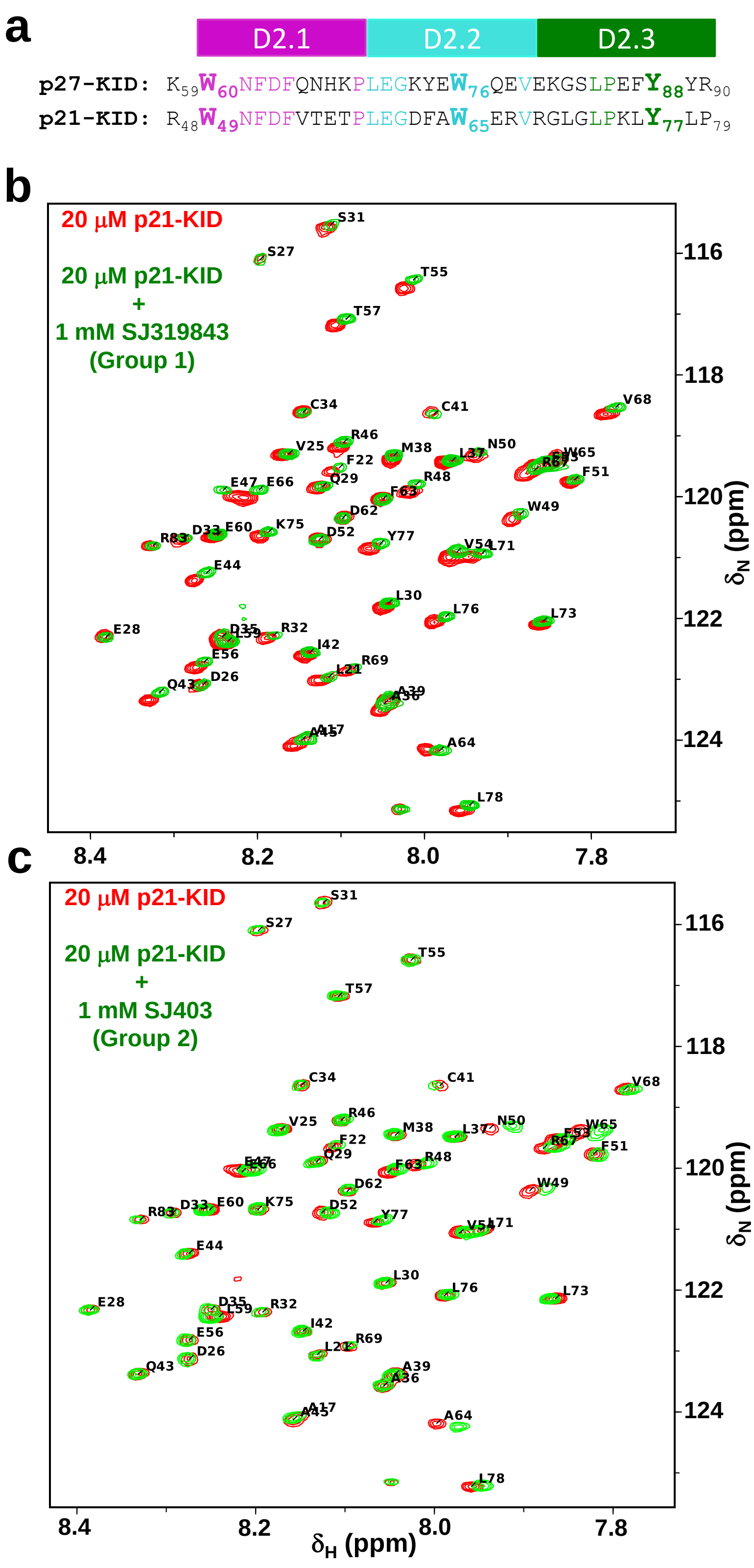

Supplementary Figure 6. (**a**) Comparison of amino acid sequences of the D2 subdomains of p27-KID and p21-KID**; (b, c)** Overlaid 2D ^{1}H-^{15}N HSQC NMR spectra of $^{2}H/^{15}N$-p21-KID (20 µM) in the absence (red) and presence (green) of 1 mM SJ319843 (**b,** Group 1) and 1 mM SJ403 (**c,** Group 2), respectively; glycine region was omitted for improved clarity.

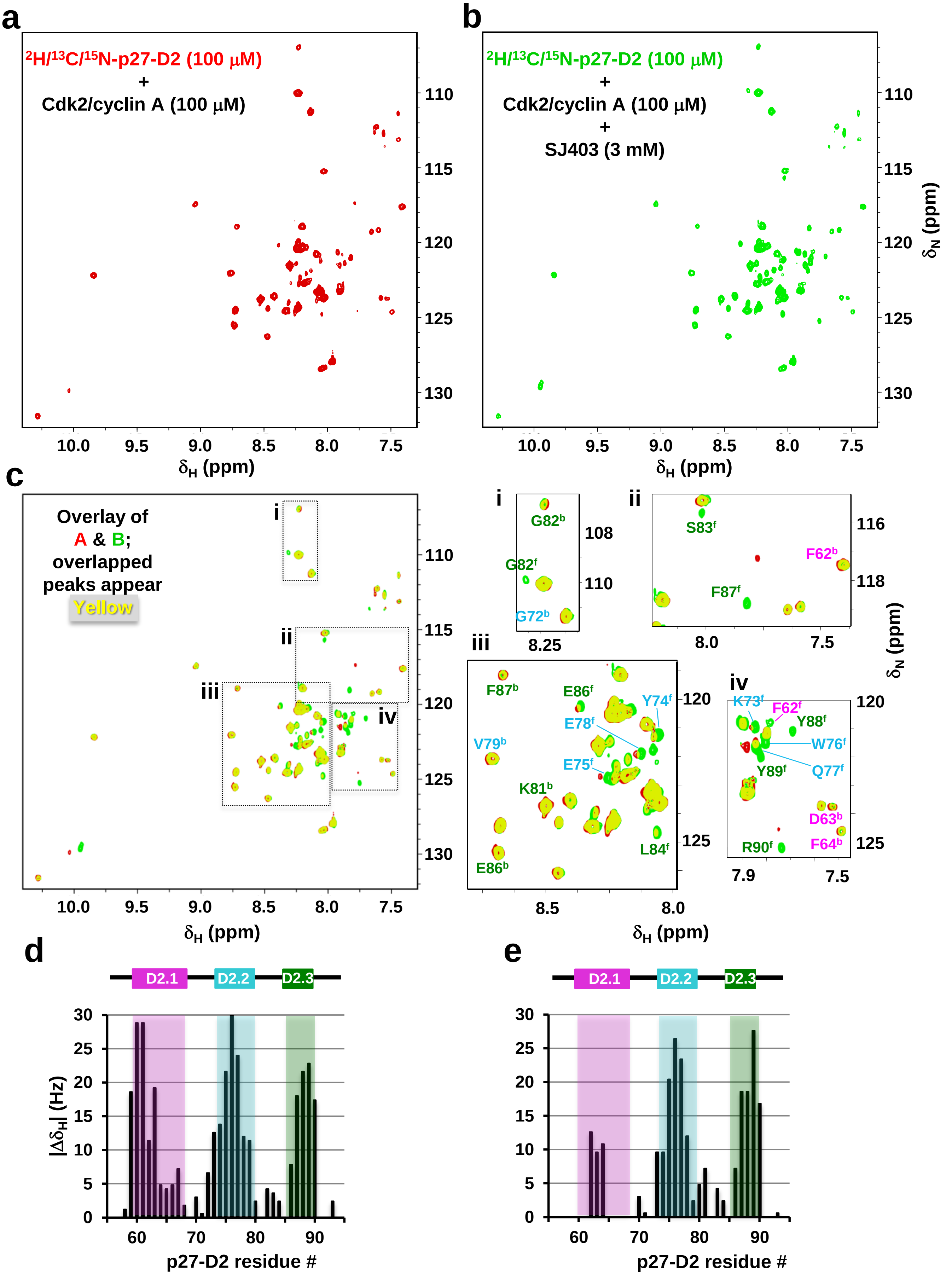
a
2H/13C/15N-p27-D2 (100 µM)
+
Cdk2/cyclin A (100 µM)
b
2H/13C/15N-p27-D2 (100 µM)
+
Cdk2/cyclin A (100 µM)
+
SJ403 (3 mM)
δH (ppm)
δN (ppm)
c
Overlay of
A & B;
overlapped
peaks appear
Yellow
i
ii
iii
iv
G82b
G82f
G72b
S83f
F62b
F87f
F87b
E86f
Y74f
E78f
V79b
E75f
K81b
E86b
L84f
K73f
F62f
Y88f
W76f
Q77f
Y89f
D63b
F64b
R90f
d
e
D2.1
D2.2
D2.3
|ΔδH| (Hz)
p27-D2 residue #

Supplementary Figure 7. 2D NMR analysis of the displacement of $^{2}H/^{13}C/^{15}N$-p27-D2 from Cdk2/cyclin A by SJ403. 2D ^{1}H-^{15}N TROSY-HSQC spectra of $^{2}H/^{13}C/^{15}N$-p27-D2/Cdk2/cyclin A in the absence (**a**) and presence of 3 mM SJ403 (**b**). In (**c**), the spectra in (**a**) and (**b**) are overlaid to emphasize resonance perturbations associated with the binding of SJ403 to p27-D2. Unperturbed resonances appear in yellow color. Examples of resonances of p27-D2 bound to Cdk2/cyclin A that decreased in intensity upon interaction with SJ403 are labeled with a superscripted "b". Examples of resonances corresponding to free p27-D2 that increased in intensity in the presence of SJ403 are marked by a superscripted "f". (**d, e**) Chemical shift perturbations observed for resonances of isolated ^{15}N-p27-D2 in the presence of 3 mM SJ403 (**d**) and for the population of free p27-D2 observed for the sample containing $^{2}H/^{13}C/^{15}N$-p27-D2, Cdk2/cyclin A, and 3 mM SJ403. The locations of sub-domains within p27-D2 are indicated by the shaded boxes.

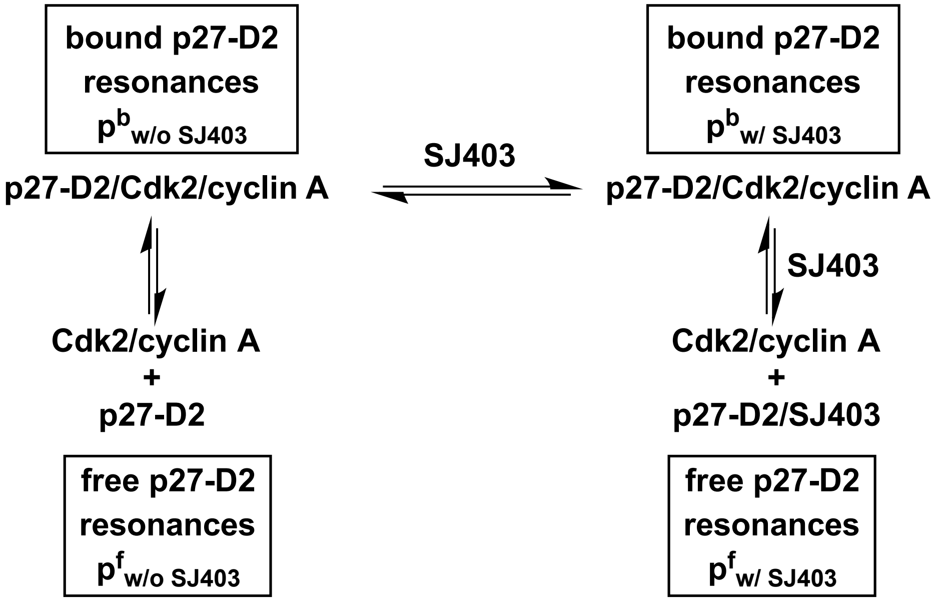

Supplementary Figure 8. Graphic representation of the displacement of p27-D2 by SJ403. The initial equilibrium is perturbed by the addition of SJ403, resulting in increased population of free p27-D2, which is bound to the small molecule, SJ403.

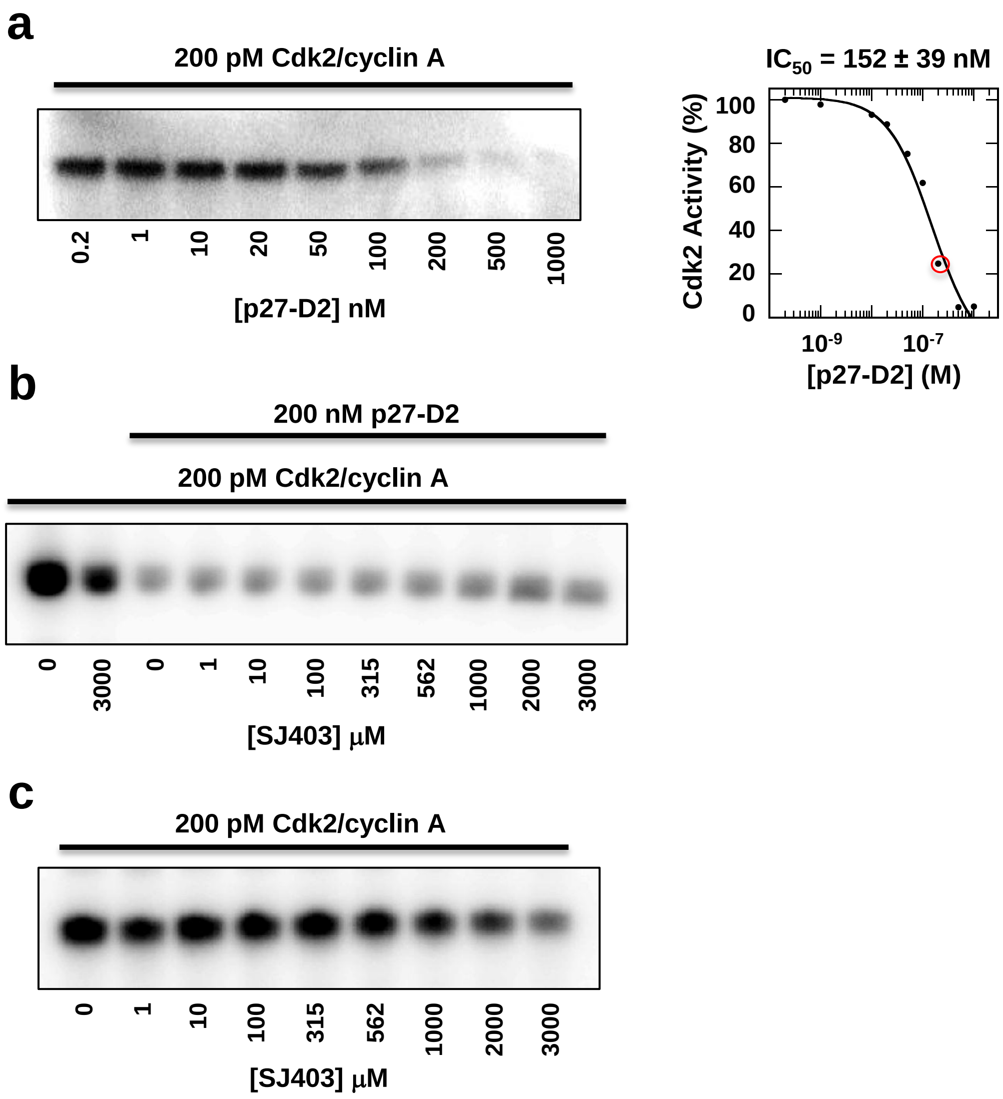

Supplementary Figure 9. Results of Cdk2 activity assays for Cdk2/cyclin A (200 pM) in the presence of (**a**) increasing concentrations of p27-D2, (**b**) p27-D2 (200 nM) and increasing concentrations of SJ403, and (**c**) increasing concentrations of SJ403. The panels show phosphoimager results after SDS-PAGE analysis of ^{32}P incorporation from ATP into the substrate, Histone H1. A single set of representative results are shown; all experiments were performed in triplicate.

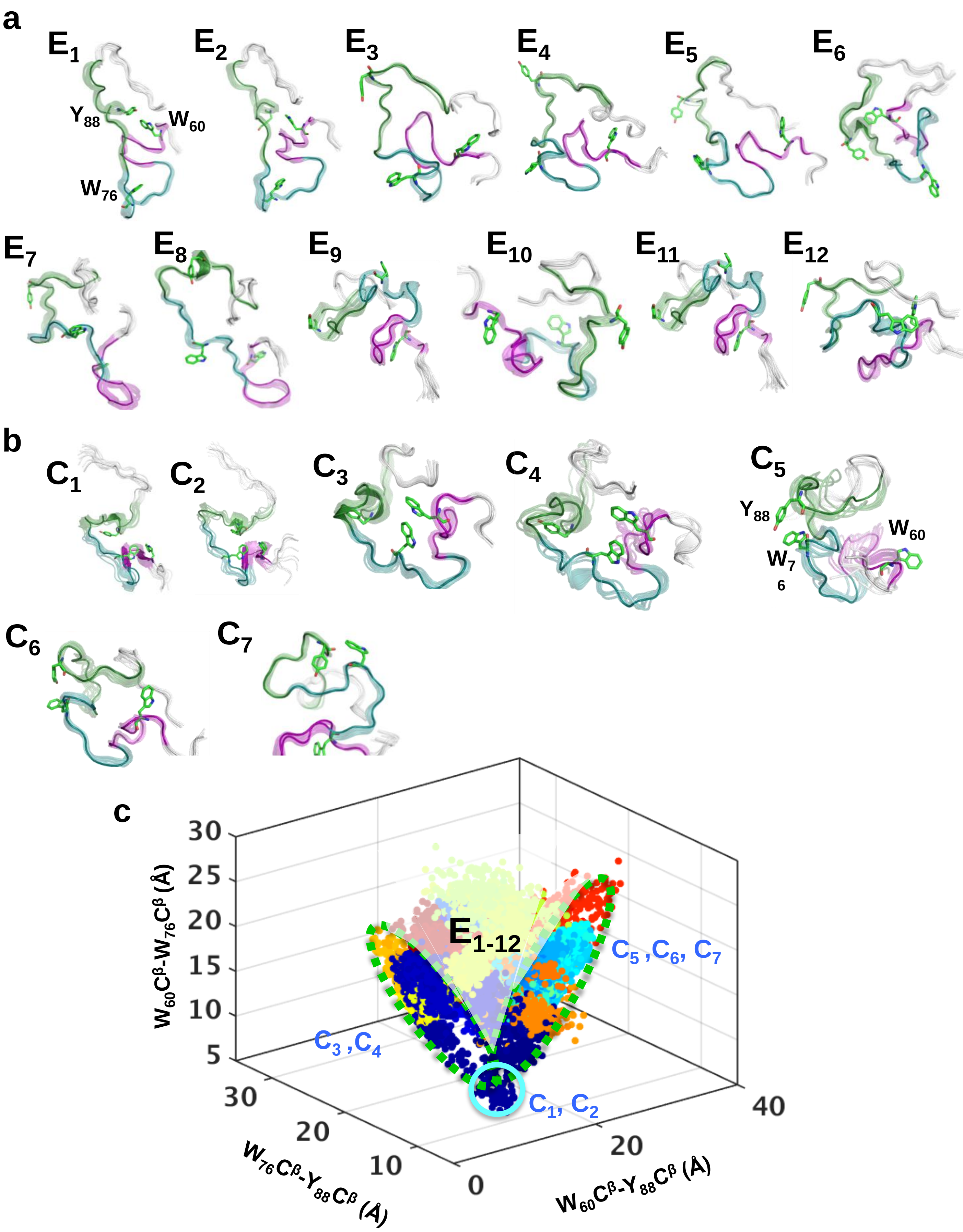
a
E_1
E_2
E_3
E_4
E_5
E_6
Y_{88}
W_{60}
W_{76}
E_7
E_8
E_9
E_{10}
E_{11}
E_{12}
b
C_1
C_2
C_3
C_4
C_5
Y_{88}
W_{60}
W_7
6
C_6
C_7
c
E_{1-12}
C_5, C_6, C_7
C_3, C_4
C_1, C_2
$W_{60}C^{\beta}$-$W_{76}C^{\beta}$ (Å)
30
25
20
15
10
5
30
20
10
0
20
40
$W_{76}C^{\beta}$-$Y_{88}C^{\beta}$ (Å)
$W_{60}C^{\beta}$-$Y_{88}C^{\beta}$ (Å)

Supplementary Figure 10. Compact (**C**; panels **a**) and Extended (**E**; panel **b**) conformations from the p27-D2 MD simulation partitioned using both the time-series information and distance distributions from Fig. 9. The times at which these representative structures were selected are indicated in Fig. 9a. The atoms of W_{60}, W_{76} and Y_{88} are illustrated as sticks in each structure. The blurring represents the conformations of molecules within 0.1 ns before and after the sampled time in the trajectory. Panel (**c**) summarizes the distribution of conformers along the distances between W_{60}-W_{76} Cβ, W_{60}-Y_{88} Cβ and W_{76}-Y_{88} Cβ atoms. Each conformer is colored according to its timestamp from the simulation, with the color drawn uniformly from fifty bins between 0 (blue) microseconds and 0.4 (red) microseconds. The conformers from the respective compact (**C**) states are shown as three ellipses. The first ellipse (light blue color) represents conformers from **C1** and **C2**, where the distance between W_{60}-Y_{88} Cβ and W_{76}-Y_{88} Cβ atoms were small. The conformers from **C3-7** (marked by green ellipses) are shown in regions where either the W_{60}-Y_{88} Cβ distance was small (and the W_{76}-Y_{88} Cβ distance was large) or vice-versa. The extended (**E**) conformers are highlighted in **E1-E12**, which were taken from other regions of the simulation (Fig. 9a).

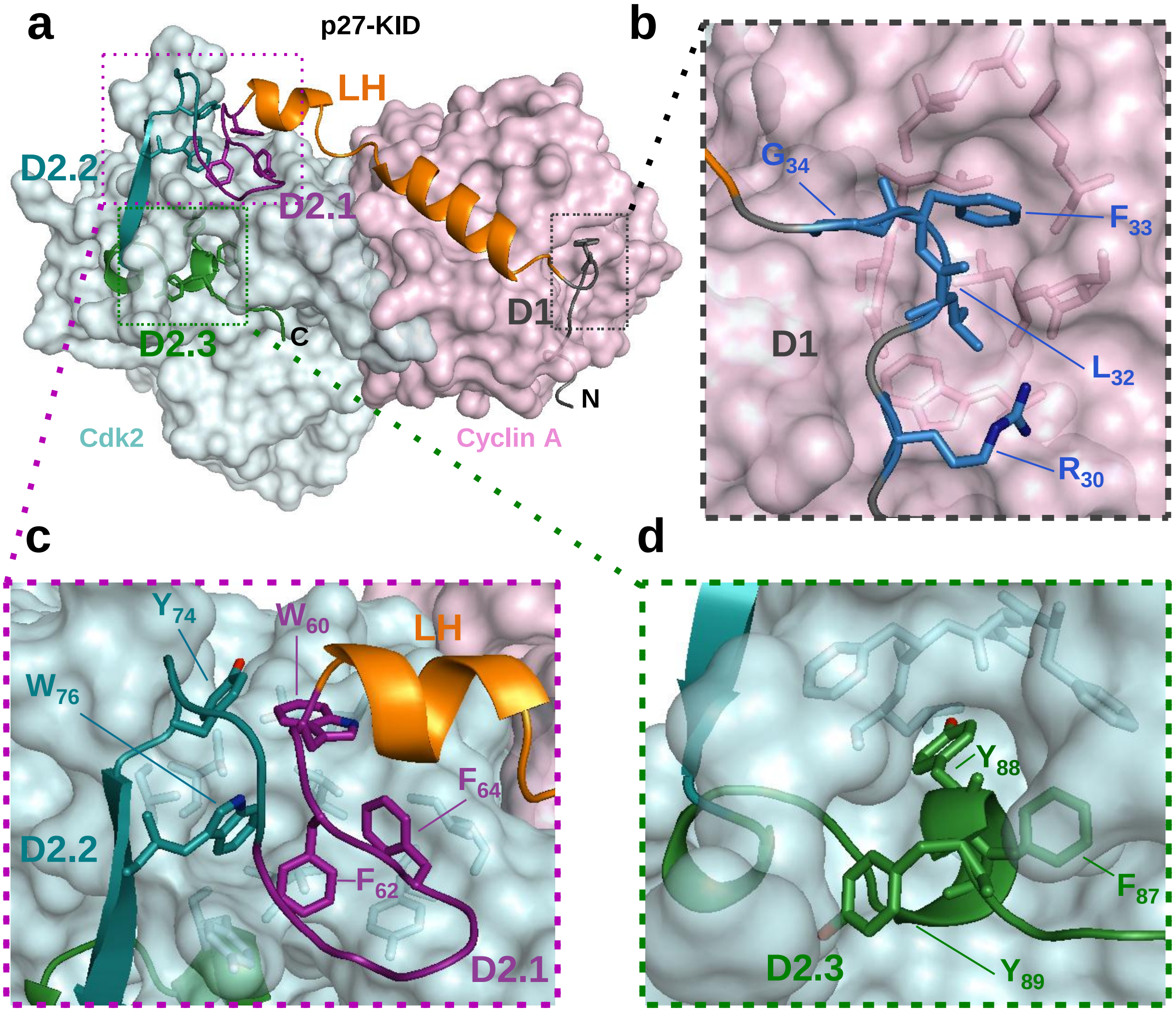

Supplementary Figure 11. Illustration of the structure of aromatic residues within sub-domain D2 and key binding residues with sub-domain D1 of p27 from the p27-KID/Cdk2/cyclin A structure. (**a**) View of entire structure. (**b–d**) Expanded views of sub-domain D1 binding to cyclin A (**b**) and of the binding of sub-region D2.1-D2.2 (**c**) and D2.3 (**d**) to Cdk2.

a

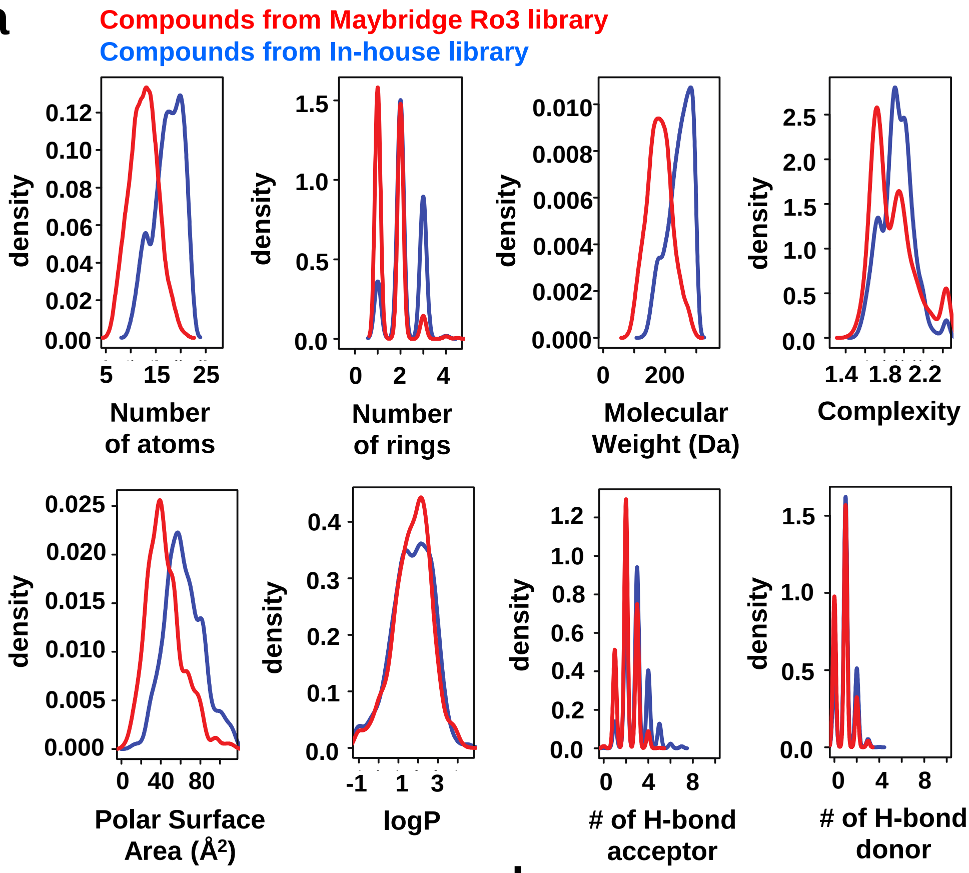

b

p27-KID

GSHM-E$_{22}$HPKPSACRNLFGPVDHEELTRDLEKHCRDME
EASQRKWNFDFQNHKPLEGKYEWQEVEKGSLPE
FYYRPPRPPKGACKVPAQE$_{105}$

d

p27-D2

GSHM-R$_{58}$KWNFDFQNHKPLEGKYEWQEVEKGSLPE
FYYRPPRPPKGACKVPAQE$_{105}$

c

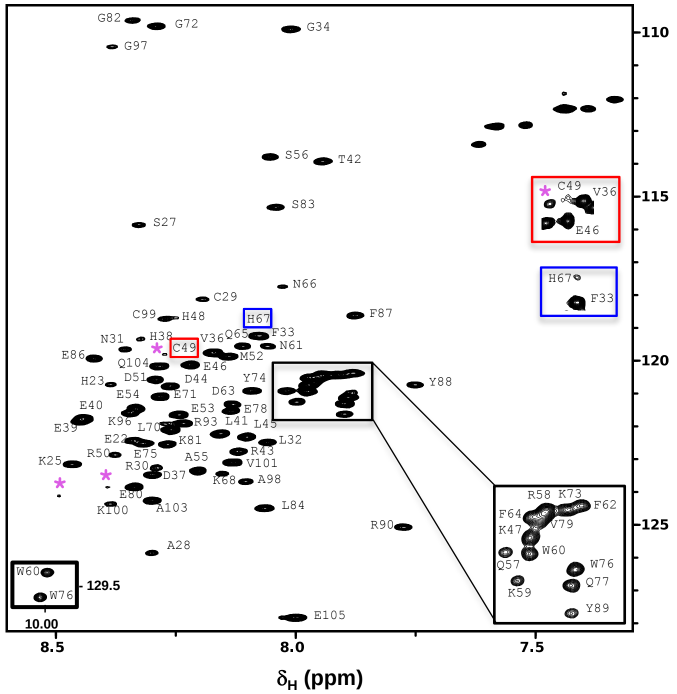

e

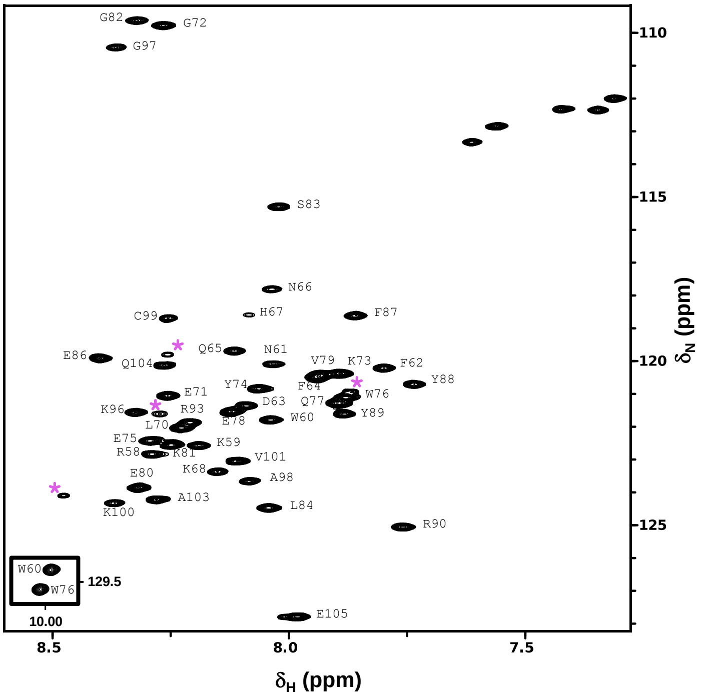

Supplementary Figure 12. (a) Chemical features of molecules in the two fragment libraries used to screen for binding to p27-KID. Features of the Maybridge Ro3 library are shown in red and those of the **In-house** fragment library in blue. **(b-e)** Amino acid sequences and 2D ^{1}H-^{15}N HSQC spectra (showing resonance assignments) for p27-KID (**b, c**) and p27-D2 (**d, e**), respectively. Puple asterisk (*) indicate resonances arising from minor conformers. In (**c**), the regions marked by colored boxes are expanded and shown at a lower contour level in the insets at the right.

a

ID	Chemical structure	Binding Group	AlogP	tPSA	H-bond acceptor	H-bond donor	MW (Da)
CC34414	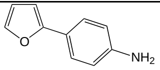	Group 1	2.19	35.25	2	2	159.18
SJ000053521	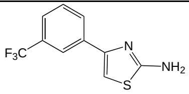	Group 1	2.97	67.14	3	2	244.24
SJ000483069	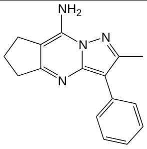	Group 1	2.76	56.21	4	2	264.32
SJ000023358	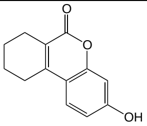	Group 1	3.09	46.53	3	1	216.23
SJ000572774	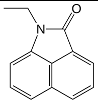	Group 1	2.24	20.31	2	0	197.23
SJ000248846	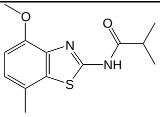	Group 1	3.17	79.45	4	1	264.34
SJ000572486	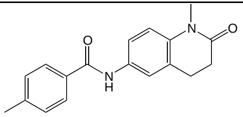	Group 1	2.75	49.41	4	1	294.34
SJ000319768	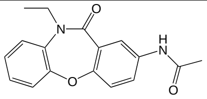	Group 1	2.02	58.64	5	1	296.32
SJ000208978	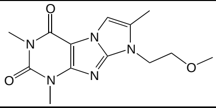	Group 2	0.07	72.08	8	0	291.30

Supplementary Table 1. Names, chemical structure, Group designation, and chemical parameters for molecules identified as “hits” in the initial fragment library screen (**a**), in the follow-up screen of chemically similar compounds in the same libraries (**b**), and from the in-house high-throughput screening library identified on the basis of the field alignment models (**c**).

b

ID	Chemical structure	Binding mode	AlogP	tPSA	H-bond acceptor	H-bond donor	MW (Da)
SJ000572513	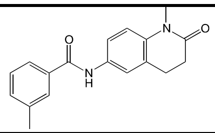	Group 1	2.75	49.41	4	1	294.34
SJ000572710	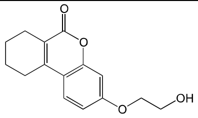	Group 1	2.77	55.76	4	1	260.28
SJ000319843	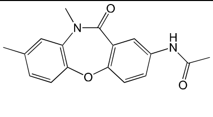	Group 1	2.15	58.64	5	1	296.32
SJ000319683	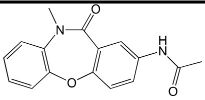	Group 1	1.67	58.64	5	1	282.29
SJ000572487	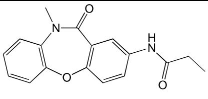	Group 1	2.33	58.64	5	1	296.32
SJ000572443	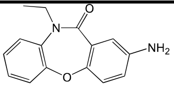	Group 1	2.15	55.56	4	2	254.28
SJ000572401	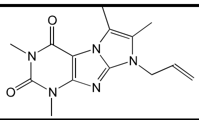	Group 2	1.1	62.85	7	0	287.31
SJ000572403	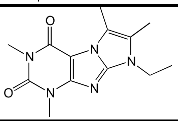	Group 2	0.83	62.85	7	0	275.30
SJ000572405	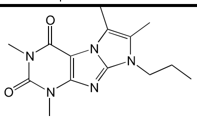	Group 2	1.35	62.85	7	0	289.33
SJ000572407	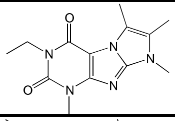	Group 2	0.83	62.85	7	0	275.30
SJ000572409	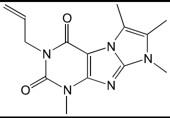	Group 2	1.1	62.85	7	0	287.31
SJ000572542	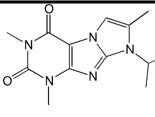	Group 2	0.92	62.85	7	0	275.30
SJ000852806	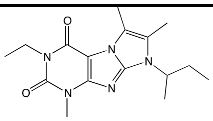	Group 2	2.08	62.85	7	0	317.39
SJ000271822	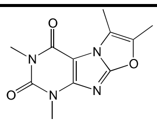	Group 2	0.285	71.06	7	0	248.24

Supplementary Table 1 (continued)

c

ID	Chemical structure	Binding Group	AlogP	tPSA	H-bond acceptor	H-bond donor	MW (Da)
SJ000017408	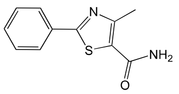	Group 1	1.79	84.22	3	2	218.27
SJ000023360	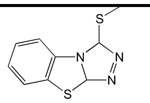	Group 1	3.05	83.72	3	0	221.3
SJ000023362	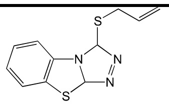	Group 1	3.664	83.72	3	0	247.34
SJ000024853	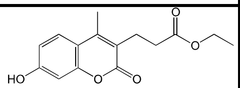	Group 1	2.76	72.83	5	1	276.28
SJ000034522	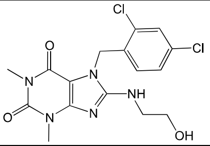	Group 1	2.623	90.69	8	2	298.24
SJ000039342	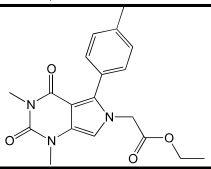	Group 1	2.63	71.84	7	0	355.39
SJ000039843	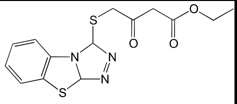	Group 1	3.01	127.1	6	0	355.4
SJ000045744	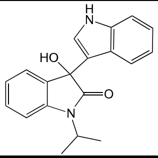	Group 1	2.94	56.33	4	2	306.36
SJ000013100	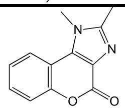	Group 2	2.02	44.12	4	0	214.22
SJ000082958	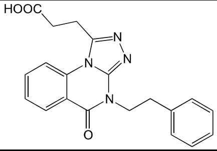	Group 2	2.69	88.32	7	1	362.38
SJ000572844	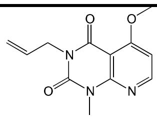	Group 2	0.97	62.74	6	0	247.25
SJ000852808	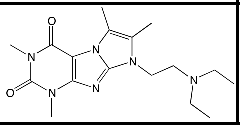	Group 2	1.317	66.09	8	0	346.43

Supplementary Table 1 (continued)
